# Antioxidant Defense Enzyme Genes and Asthma Susceptibility: Gender-Specific Effects and Heterogeneity in Gene-Gene Interactions between Pathogenetic Variants of the Disease

**DOI:** 10.1155/2014/708903

**Published:** 2014-05-05

**Authors:** Alexey V. Polonikov, Vladimir P. Ivanov, Alexey D. Bogomazov, Maxim B. Freidin, Thomas Illig, Maria A. Solodilova

**Affiliations:** ^1^Department of Biology, Medical Genetics and Ecology, Kursk State Medical University, 3 Karl Marx Street, Kursk 305041, Russia; ^2^Department of Pediatrics, Kursk State Medical University, 11a Koltsov Street, Kursk 305035, Russia; ^3^Research Institute for Medical Genetics, Siberian Branch of Russian Academy of Medical Sciences, 10 Nabereznaya Ushaiki Tomsk 634050, Russia; ^4^Research Unit of Molecular Epidemiology, Helmholtz Zentrum München, German Research Center for Environmental Health, Ingolstädter Landstraße 1, 85764 Neuherberg, Germany; ^5^Hanover Unified Biobank, Hanover Medical School, Carl-Neuberg-Strasse 1, 30625 Hanover, Germany

## Abstract

Oxidative stress resulting from an increased amount of reactive oxygen species and an imbalance between oxidants and antioxidants plays an important role in the pathogenesis of asthma. The present study tested the hypothesis that genetic susceptibility to allergic and nonallergic variants of asthma is determined by complex interactions between genes encoding antioxidant defense enzymes (ADE). We carried out a comprehensive analysis of the associations between adult asthma and 46 single nucleotide polymorphisms of 34 ADE genes and 12 other candidate genes of asthma in Russian population using set association analysis and multifactor dimensionality reduction approaches. We found for the first time epistatic interactions between ADE genes underlying asthma susceptibility and the genetic heterogeneity between allergic and nonallergic variants of the disease. We identified *GSR* (glutathione reductase) and *PON2* (paraoxonase 2) as novel candidate genes for asthma susceptibility. We observed gender-specific effects of ADE genes on the risk of asthma. The results of the study demonstrate complexity and diversity of interactions between genes involved in oxidative stress underlying susceptibility to allergic and nonallergic asthma.

## 1. Introduction

Bronchial asthma (BA) is a common chronic inflammatory disease of the airways characterized by variable and recurring symptoms, reversible airflow obstruction, and bronchospasm [1]. There is a considerable body of evidence demonstrating that asthma is a multifactorial disease which results from complex interactions between susceptibility genes of small-to-modest effects and equally important environmental factors [2, 3].

In the recent years, the relationships between common genetic variants and BA risk are being reported with rapidly increasing frequency. Large-scale genome-wide association studies (GWAS) have been recently done to look for asthma susceptibility genes in ethnically diverse populations of the world [4, 5]. However, the findings obtained by GWAS were limited by the strongest associations of a few number of genetic variants that achieved genome-wide significance level. In addition, the genome-wide associations are found to be quite difficult to interpret with respect to disease pathogenesis [4–7]. Meanwhile, hundreds to thousands of genetic markers associated with a disease risk are not interpreted because they have not achieved the genome-wide significance level, thus accounting for the “missing heritability” of complex diseases [8]. This means that GWAS approach is powerless in the detecting genes of small-to-modest effects which represent a polygenic background of multifactorial disease. Additionally, genetic diversity of human populations [9], heterogeneity of disease pathogenesis [10], and especially a complexity of gene-gene interactions [11, 12] complicate our opportunities in unraveling the molecular mechanisms underlying complex diseases including asthma.

It is widely agreed that the expression of a disease phenotype may not accurately be predicted from the knowledge of the effects of individual genes because of complex nonlinear interactions between genes, including epistatic and additive interactions [8, 13]. To address this issue, several powerful data-mining approaches have been developed to identify susceptibility genes involved in such complex interactions [14–16]. One of them, multifactor dimensionality reduction (MDR) method, was developed to reduce the dimensionality of multilocus information to improve the ability to detect genetic combinations that confer disease risk in relatively small samples [16, 17]. With set association analysis (SAA), contributions from multiple SNPs are combined by forming a sum of single-marker statistics, which results in a single genome-wide test statistic with high power [14].

An important task for a genetic epidemiologist utilizing a candidate gene approach is the selection of appropriate genes and SNPs for testing the disease association. Compared with studying individual genes, the inferences derived from a hypothesis-driven candidate pathway study are enhanced by allowing global conclusions about the involvement of entire biochemical pathway to the pathogenesis of disease [18]. Following this approach in our previous study [19], we pointed out the potential relevance of toxicogenomic mechanisms of BA in the modern world and proposed that genes for xenobiotic-metabolizing enzymes would be the most appropriate candidate genes for asthma susceptibility whose effects on the disease risk can be associated with exposure to air pollution. Due to the fact that air pollutants are the sources of reactive oxygen species (ROS), genes involved in oxidative stress can potentiate harmful effects of xenobiotics on the respiratory system [20, 21].

It is well known that oxidative stress resulting from an increased amount of ROS and an imbalance between oxidants and antioxidants plays a role in the molecular mechanisms underlying BA [22–24]. We have demonstrated that genes of antioxidant defense enzymes (ADE), such as glutamate cysteine ligase (*GCLM*) [25], glutathione peroxidase (*GPX1*) [26], catalase (*CAT*) [27], myeloperoxidase (*MPO*) [28], NADPH oxidase (*CYBA, *p22phox subunit) [29, 30], NAD(P)H: quinone oxidoreductase type 1 (*NQO1*) [31] and microsomal epoxide hydrolase (*EPHX1*) [19], are important determinants of genetic susceptibility to asthma in Russians. In the present study, we tested the hypothesis that genetic susceptibility to both allergic and nonallergic asthma is determined by complex interactions between genes involved in oxidative stress. We performed for the first time a comprehensive analysis of genomic interactions between 34 ADE genes and 12 other candidate genes in order to identify gene-gene interactions in redox homeostasis underlying polygenic mechanisms of BA.

## 2. Materials and Methods

### 2.1. Study Population

The study protocol was approved by the Ethical Review Committee of Kursk State Medical University, and written informed consent was obtained from each participant before the study. The participants comprised a total of 429 unrelated individuals (215 patients with asthma and 214 healthy controls); all are ethnically Russians from Central Russia (mainly from the Kursk region). All study subjects were recruited from the Division of Pulmonology at the Kursk Regional Clinical Hospital between 2003 and 2004. Asthma was diagnosed by qualified pulmonologists on the basis of the WHO criteria, as described previously [19, 32]. The mean age of the patients with asthma (94 men and 121 women) was 43.3 years (ranging from 16 to 67 years), and the mean age of the healthy subjects (105 men and 109 women) was 41.3 years (ranging from 17 to 84 years). Skin prick tests were conducted and total serum IgE levels were determined in all study subjects. Patients with positive skin prick tests and high level of total IgE were defined as patients with allergic asthma (64 men and 92 women). Asthmatics who showed either negative skin prick test results (wheal size: <5 mm) or a normal total IgE level (<0.35 IU) were considered to be patients with nonallergic asthma (29 men and 27 women). Data on allergic status were not available for three asthmatics. A strong positive family history of asthma was found in the case group (40.1%) in comparison with controls (6.7%).

### 2.2. Selection of Candidate Genes

The candidate genes for this study were selected according to the guidelines for genetic association studies proposed by Cooper and coauthors [33]. We used the following criteria to select ADE genes and their genetic polymorphisms satisfying our study's purposes: (1) enzymes should represent key players involved in the regulation of redox processes; (2) enzymes should cover all biochemical pathways of redox homeostasis entirely, including enzymes possessing antioxidant activity (*GPX1*,* SOD2*,* CAT*,* GSTM1*, etc.) and those with prooxidant activity (i.e., ROS-generating enzymes such as CYBA, MPO, and CYP2E1); (3) enzymes should be expressed in the lung and/or airways (the expression patterns of the selected ADE genes in human tissues and organs are shown in Supplementary Material available online at http://dx.doi.org/10.1155/2014/708903); (4) SNPs should be functionally significant, whenever possible; and (5) minor allele frequency should be more than 5%. Following these criteria, 34 polymorphisms of 24 ADE genes have been selected from published literature and public databases. 

### 2.3. DNA Extraction and Genotyping

Genomic DNA of all study participants was isolated from 5–10 mL of peripheral blood samples, collected in K3-EDTA tubes by venipuncture, and maintained at −20°C until processed. Twenty-five of the selected gene polymorphisms had been genotyped in our previous studies [19, 25–31]. In the present study, another nine ADE gene polymorphisms such as* GPX2* (rs17880492),* GPX3* (rs2070593)* GPX4 *(rs713041),* GSR* (rs2551715),* SOD2* (rs4880),* SOD3* (rs2536512),* PRDX1* (rs17522918),* TXNRD1* (rs1128446), and* FMO3* (rs2266782) have been genotyped. Additionally, we genotyped 12 polymorphisms of 9 candidate genes of asthma such as* TNF* (rs1800629),* IL1B *(rs16944),* IL3* (rs40401 and rs31480),* IL5 *(rs2069812),* CSF2RB* (rs131840),* IL9* (rs2069885),* SCGB1A1* (rs11549442), and* SERPINA1* (rs17580, rs143370956, and rs11568814). Majority of them have been reported to be associated with the risk of asthma and/or asthma-related phenotypes in Russians [34–37]. A complete list of 46 studied SNPs is given in Table 1. Genotyping of the selected polymorphisms was done using restriction fragment length polymorphism assays according to the published protocols (genotyping protocols are available upon request). All of the genotyping was done blindly to the case-control status and the repeatability test was conducted for the 5% of total subjects, resulting in a 100% concordance rate.

### 2.4. Statistical Analysis

The concordance of genotypes prevalence in patients with asthma and healthy controls with values expected under Hardy-Weinberg equilibrium was assessed by Pearson's chi-square test. The association between ADE gene polymorphisms and asthma was examined with binary logistic regression analysis with calculation of odds ratios (OR) and 95% confidence intervals (CI). The statistical calculations were done using Statistica for Windows (v8.0) software package (StatSoft; Tulsa, OK, USA). The statistical significance was established at the *P* ≤ 0.05 level. Bonferroni correction for *P* values (*P*_adj_) was applied in cases when multiple tests were performed.

Two bioinformatic approaches, SAA and MDR, were applied for the analysis of gene-gene interactions. The principle of SAA is described in detail elsewhere [14, 55] and implemented in a statistical program SUMSTAT (http://linkage.rockefeller.edu/ott/sumstat.html/). Briefly, the method combines the information derived from measurements of allelic/genotype association and departure from Hardy-Weinberg equilibrium into a single, genome-wide statistic. The markers with high Hardy-Weinberg disequilibrium (HWD) values in the control group are trimmed and are not considered for further analysis. For the remaining markers, effects of allelic/genotype association with disease and HWD values are then combined into a single* Sum* statistic [56]. *P* values reported by the program were calculated by permutations. The number of permutation tests was set at 10000.

MDR is a flexible nonparametric and genetic model free method for analysis of high-order nonlinear or nonadditive gene-gene interactions [15]. The method has been proposed to overcome limitation of logistic regression which deals with many factors simultaneously and fails to characterize epistatic models in the absence of main effects, due to the hierarchical model-building process leading to an increase in type II errors and decreased power [17]. The MDR method uses a constructive induction algorithm that converts two or more variables such as SNPs into a single attribute. In particular, SNPs are pooled into high and low risk group, effectively reducing the multifactor prediction from *n* dimension to one dimension. Best models for each locus combination are selected by repeating the analysis for up to 10 seeds after shuffling the order of individuals and applying 10-fold cross-validation each time. Average of cross-validation consistency (CVC) together with training and test accuracy is calculated for each locus combination. CVC is defined as the number of times a particular interaction model is selected across 10 cross-validation datasets. We performed statistical calculations using MDR software (http://www.multifactordimensionalityreduction.org/). Statistical significance of the best models selected for each SNP combination was determined using 1000-fold permutation testing. The significance of the final MDR model was determined empirically by 1000 permutations using the Monte-Carlo procedure implemented into the MDRpt software (http://sourceforge.net/projects/mdr/). *P* values for CVC were considered statistically significant at ≤0.05 levels. To visualize and interpret the results obtained from MDR, we used interaction dendrograms. 

Both the SAA and MDR are limited by the identification of a few number of high penetrance interacting genes, whereas a larger portion of genes of low-to-moderate effects remain out of the analysis. To address this issue, we performed post hoc comparisons of two-locus genotype combinations (only for those SNPs which were found in gene-gene interaction models obtained by SAA and/or MDR methods) between the case and control groups to look for the genotype combinations which determine the risk of asthma. The observed associations were adjusted for multiple tests using Bonferroni procedure.

## 3. Results

### 3.1. Allele and Genotype Frequencies in Asthmatics and Controls

Allele and genotype frequencies of the studied genes are shown in Tables 2 and 3, respectively. After adjusting for multiple tests, the only statistically significant association was found between the* IL5 *C-703T polymorphism and BA. The −703CC genotype was found to be associated with the risk of allergic asthma (OR = 0.44; 95% CI 0.29–0.67; *P* = 0.0001  (*P*_adj_) = 0.004). In gender-specific analysis, this association was seen in both men (OR = 0.50; 95% CI 0.27–0.94; *P* = 0.03) and women (OR = 0.40; 95% CI 0.22–0.70; *P* = 0.001) but did not reach a statistical significance after Bonferroni correction for multiple tests (*P* > 0.05). No association of this genotype was found with nonallergic asthma in both sexes. 

### 3.2. Gene-Gene Interactions in Asthma Revealed by Set Association Analysis

**Figure 1:**
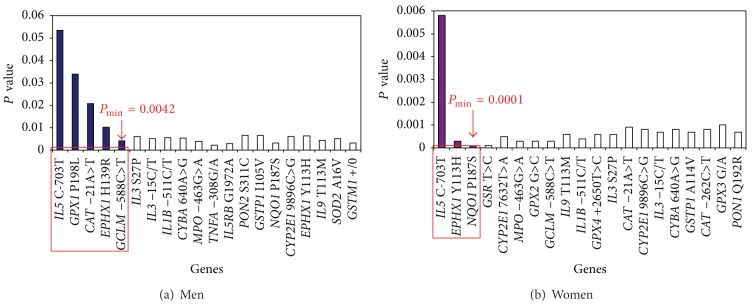
The results of statistical modeling of gene-gene interactions in allergic asthma using set association approach. Significance level of *S*_*n*_ statistic as a function of the number *n* of SNPs in different genes which are included at each step for gene-gene interactions analysis. The smallest significance level, *P*_min⁡_, occurs with 5 SNPs in males and with** 3** SNPs in females. The interacting genes in the models are circled in red.

**Figure 2:**
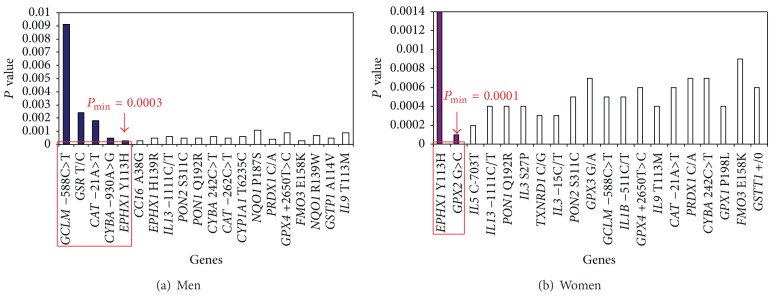
The results of statistical modeling of gene-gene interactions in nonallergic asthma using set association approach. Significance level of *S*_*n*_ statistic as a function of the number *n* of SNPs in different genes which are included at each step for gene-gene interactions analysis. The smallest significance level, *P*_min⁡_, occurs with 5 SNPs in males and with 2 SNPs in females. The interacting genes in the models are circled in red.

Taking into account the polygenic basis of asthma, it was an important task to investigate high-order gene-gene interactions using specialized bioinformatics approach called set association analysis which captures the simultaneous effects of multiple genes and achieves a global view of gene action and interaction [14, 55]. For trimming, we considered values of departures from HWE (HWD values) exceeding the 99th percentile of chi-square (*χ* ^2^ ≥ 6.6, *df* = 1) in the control group [14]. There were no HWD values larger than 6.6 in the control subjects, so the trimming procedure to our dataset was avoided. To calculate single-locus test statistics, we used the difference in the distribution of genotypes for the *i*th SNP between cases and controls (chi-square test for 2 × 3 tables). We tested up to *N* = 46 sums (*S*_*n*_) in allergic and nonallergic asthma, separately in men and women. When we added more SNPs to the *S*, *P* values tended to increase; that is, adding additional markers introduces noise to the *S*. In allergic asthma, the smallest *P* values were obtained for a sum of 5 SNPs (the* GPX1 *P198L,* CAT* −21A>T,* EPHX1 *H139R,* GCLM* −588C>T, and* IL5 *C-703T) for men and a sum of 3 SNPs (the* EPHX1 *Y113H,* NQO1* R139W, and* IL5 *C-703T) for women (Figure 1). After 1000 permutation tests, the global significance levels (*P*_min⁡_) of 0.0042 for men and 0.0001 for women were obtained. In nonallergic asthma, the smallest significance levels appeared for 5 SNPs (the* GCLM* −588C>T,* GSR *T>C,* CAT* −21A>T,* CYBA* −930A>G, and* EPHX1 *Y113H) in men and for 2 SNPs (the* EPHX1 *Y113H and* GPX2 *G>A) in women (Figure 2). The global significance levels of 0.0003 for men and 0.0001 for women were obtained.

### 3.3. Modeling for Gene-Gene Interactions in Asthma Using MDR Method

The MDR method was used for a purpose of modeling gene-gene interactions underlying allergic and nonallergic asthma in men and women. Firstly, we used an exhaustive search algorithm to evaluate all interactions among all possible subsets of the polymorphisms.  Table 4 shows the cross-validation consistency and the prediction error for gene-gene interactions (from two- to four-locus interactions) obtained from MDR analysis in both allergic and nonallergic asthma. The only statistically significant (empirical *P* = 0.001) three-locus model involving interactions between* EPHX1* Y113H,* IL5* C-703T, and* GPX1 *P198L loci was discovered. The model had a minimum prediction error of 40.9 and a maximum cross-validation consistency of 50% in allergic asthma in women (*P*_min⁡_ = 0.001). None of the rest *n*-locus models in both allergic and nonallergic asthma showed a statistical significance in the MDR analysis using an exhaustive search algorithm, thereby motivating us to apply a forced search algorithm for further MDR analyses in order to build the best *n*-locus models in men with allergic asthma and in both sexes with nonallergic asthma. Following this approach, we obtained one statistically significant (empirical *P* = 0.001) four-locus model comprising interactions between* CAT* −21A>T,* GPX2 *G>A,* GSR T>C*, and* IL5* C-703T in men with allergic asthma. The model had a minimum prediction error of 26.8 and a maximum cross-validation consistency of 100%.

**Figure 3:**
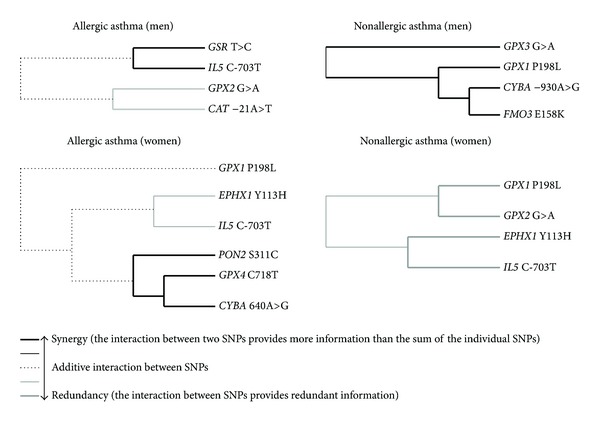
Dendrograms of gene-gene interactions in the pathogenetic variants of asthma (MDR method). Dendrograms show both complexity and diversity of interactions between polymorphic genes of antioxidant defense enzymes in allergic and nonallergic asthma (dendrograms are stratified by gender). Each dendrogram comprises a spectrum of lines representing a continuum from synergy (black) to redundancy (gray) of gene-gene interactions. The lines range from bold black, representing a high degree of synergy (positive information gain), thin black, representing a lesser degree, and dotted line representing the midway point between synergy and redundancy. On the redundancy end of the spectrum, the highest degree is represented by bold gray (negative information gain) with a lesser degree represented by thin gray.

Figure 3 shows the dendrograms illustrating high-order gene-gene interactions between the ADE loci in the pathogenetic variants of asthma in men and women. According to the figure, there is a strong difference in the structure of gene-gene interactions between men and women. In particular, synergistic interaction effect was found between the* GSR T>C* and* IL5* C-703T loci in men. Moreover, the* CAT* −21A>T and* GPX2* G>A gene polymorphisms had a strong antagonistic effect on the risk of allergic asthma in men. On the contrary, the hierarchical cluster analysis of the MDR data in women showed that the* CYBA* 640A>G,* GPX4* C718T, and* PON2* S311C gene polymorphisms have a strong synergistic interaction effect on the risk of allergic asthma. The* EPHX1* Y113H and* IL5* C-703T SNPs had a moderate antagonistic effect on the allergic asthma risk in women. Also, a relatively independent effect of the* GPX1* P198L gene polymorphism on the risk of allergic asthma was seen.

On the next step, a forced search algorithm was applied to analyze all possible* n*-locus interactions in nonallergic asthma. The best 4-locus model including* GPX1 *P198L,* GPX3 *G/A,* CYBA* −930A>G, and* FMO3 *E158K polymorphisms was found in men (empirical *P* = 0.01). The model had a minimum prediction error of 26.1 and a cross-validation consistency of 100%. The forced MDR analysis performed in women revealed a model including* GPX1 *P198L,* GPX2* G>A,* EPHX1* Y113H, and* IL5* C-703T polymorphisms with a minimum prediction error of 28.1 and a cross-validation consistency of 100% (empirical *P* = 0.001).

### 3.4. Post Hoc Association Analysis of Two-Locus Genotype Combinations

Then, we performed a post hoc comparison of genotype frequencies between the case and control groups with a focus on those ADE genes which were present in gene-gene interaction models obtained using SAA and MDR methods. Ten and nine two-locus combinations were found to be associated with allergic asthma in men and women, respectively (Table 5). However, only one genotype combination* GPX4 *718TC ×* CYBA* 640AG achieved statistically significant inverse association with the risk of allergic asthma in women after adjustment for multiple tests (OR = 0.37; 95% CI 0.20–0.71; *P*_adj_ = 0.002). Twelve and five two-locus genotype combinations were found to be associated with the risk of nonallergic asthma in men and women, respectively (Table 6). Four two-locus genotype combinations showed statistically significant associations with nonallergic asthma in men after Bonferroni correction for multiple comparisons:* GPX1* 198PL ×* CAT* −21AA (OR = 11.45; 95% CI 2.49–52.66; *P*_adj_ = 0.001),* GSR* TT ×* GCLM* −588CT (OR = 11.58; 95% CI 3.07–43.72; *P*_adj_ = 0.0001),* CAT* −21AA ×* CYBA* −930GG (OR = 15.64; 95% CI 2.44–100.3; *P*_adj_ = 0.001), and* GCLM* −588CT ×* CYBA* −930GG (OR = 6.71; 95% CI 2.5–17.96; *P*_adj_ < 0.0001). One genotype combination* EPHX1* 113HH ×* IL5* −703CC showed a significant association with increased risk of nonallergic asthma in women (OR = 8.58; 95% CI 2.43–30.26; *P*_adj_ = 0.001).

## 4. Discussion

### 4.1. A Summary of the Study Findings

The main purpose of our study was to investigate a comprehensive contribution of ADE genes to genetic susceptibility to allergic and nonallergic variants of BA. The single-locus analysis revealed that none of the ADE genes was associated with the risk of asthma. However, using two bioinformatics approaches, we found multilocus gene-gene interactions which are associated with the risk of allergic and nonallergic asthma in men and women in a gender-specific manner. Further, post hoc analysis allowed revealing two-locus combinations of genotypes which are significantly associated with allergic and nonallergic asthma in both sexes. A majority of the susceptibility genes identified in our study represented antioxidant defense enzymes. Moreover, interactions between ADE genes varied across the pathogenetic variants of asthma and were different in men and women suggesting both genetic heterogeneity and gender-specific genetic effects in the disease susceptibility.

### 4.2. Genetic Heterogeneity of Asthma and Complexity of Genomic Interactions Underlying the Disease

The observed differences in gene-gene interactions between allergic and nonallergic variants of asthma demonstrate a genetic heterogeneity of the disease, a situation in which the same or similar phenotype of a complex disorder is caused by different susceptibility genes [57]. It is well known that genetic heterogeneity is the general feature of many common diseases [58] and may be explained at least partially by genetic differences between human populations [9]. Bronchial asthma is a typical example for complex multifactorial disease being characterized by genetic heterogeneity [59]. In fact, the models of gene-gene interactions in the pathogenetic variants of asthma overlap only partially, thereby reflecting, on the one hand, possible differences in the molecular mechanisms of allergic and nonallergic asthma and, on the other hand, the existence of shared genes that determine common susceptibility to the disease. In particular, three ADE genes such as* GSR*,* EPHX1, *and GPX1 showed significant interaction in both variants of asthma in both men and women (except for the* GPX1 *gene in nonallergic asthma in women); therefore, they can be considered as common susceptibility genes to asthma. While the* IL5* and* PON2* genes showed an association only with allergic asthma, none of the studied genetic polymorphisms was found to be associated exclusively with the risk of nonallergic asthma.

The results of gene-gene interactions analysis are consistent with observations of other genetic studies which demonstrated an importance of ADE genes for asthma pathogenesis. In particular, we confirmed a potential role in the pathogenesis of asthma for* CYBA* and* CAT* genes that was associated with asthma in Czech [60] and Canadian [61] populations, respectively. In addition, the associations of asthma with the* IL5 *C-703T polymorphism in Russians from the city of Tomsk [35] and* IL1B* −511C>T in the Canadian Asthma Primary Prevention Study [62] have been successfully replicated in our study. Our study is consistent with the observation that glutathione-S-transferase genes M1, T1, and P1 alone and in combination with other ADE genes do not play a substantial role in the development of BA [63]. We also found for the first time genetic polymorphisms of the* GSR* and* PON2* genes can be important determinants of susceptibility to asthma, but their associations need to be confirmed in independent populations. Further studies should also be focused on the analysis of gene-gene interactions to better understanding the role of ADE genes in asthma pathogenesis. In the study of Millstein et al. [16], interactions between the* NQO1*,* MPO*, and* CAT* genes have been identified in ethnically diverse cohorts of patients with childhood asthma, whereas marker-by-marker analysis did not reveal the associations of these genes with disease susceptibility. This means that marker-by-marker approach ignores the multigenic nature of BA and does not evaluate a complexity of interactions between susceptibility genes.

Comparing the results obtained by the three statistical approaches to the analysis of gene-gene interactions, we can say that, despite gender-specific effects of genotypes on the pathogenetic variants of the disease, each of the methods showed own uniqueness and efficacy in the detecting genes associated with asthma risk. In our point of view, the advantage of SAA method is its capacity in the identification of “gene dosage effects” of different sets of ADE genes on asthmatic phenotype. Meanwhile, MDR method, especially its cluster technique, was found to be powerful in the detecting high-order epistatic interactions between ADE genes and their synergic and antagonistic effects on the asthma risk. The variability in the structure of gene-gene interactions models across the pathogenetic variants of asthma can be partially explained by differences in bioinformatic approaches to the analysis of multiple genes. Apparently, a similarity in gene-gene interactions between the models obtained by the two different bioinformatic tools may be explained by strong effects of particular genes on the asthmatic phenotype. This means that strong phenotypic effects of the* CAT*,* GPX1*,* GSR*,* GCLM*,* EPHX1*,* CYBA,* and* IL5* genes (they showed the similarity between the models) may be considered as major gene effects. And, finally, a post hoc comparative analysis of the frequencies of genotype combinations was useful in the detection of ADE genes with low or moderate effects on asthma as well as the unique combinations of ADE genotypes strongly associated with disease susceptibility. In particular, the* PON2*,* GPX2*,* GPX3*,* GPX4*,* NQO1*,* FMO3*,* SOD2,* and* IL3* genes showed low or moderate effects on asthma risk and may represent a polygenic background of the disease susceptibility. Thus, the methods complemented each other and contributed to the understanding of the polygenic nature of asthma and complexity of gene-gene interactions underlying the asthmatic phenotypes. Due to the fact that there is no universal method for comprehensive analysis of gene-gene interactions in genomic epidemiology, it makes sense to use several methods, as it has been successfully applied in the present study.

The dendrograms obtained by MDR technique (Figure 3) clearly showed complex and hierarchic pattern of interactions between ADE genes constituting the polygenic basis of the pathogenetic variants of asthma. In particular, the* GSR* T>C and* IL5* C-703T genes in men and the* CYBA* 640A>G,* PON2 *C311S, and* GPX4 *C718T loci in women had the highest degree of synergy in their interactions to determine the susceptibility to allergic asthma, whereas the highest degree of synergy in gene-gene interactions in nonallergic asthma in men was found for the* CYBA* −930A>G,* FMO3 *E158K, and* GPX1* P198L loci. In contrast, a different degree of redundancy (antagonism) in gene-gene interactions was observed between the* CAT* −21A>T and* GPX2* G>A loci in men and between the* EPHX1 *Y113H and* IL5* loci C-703T in women with allergic asthma, as well as between the* GPX1* P198L,* GPX2* G>A,* EPHX1 *Y113H, and* IL5* C-703T loci in women with nonallergic asthma. Interestingly, the* EPHX1 *Y113H and* IL5* C-703T genes showed an antagonistic character of gene-gene interactions exclusively in women with both pathogenetic variants of asthma. Notably, the* EPHX1 *Y113H genotypes did show the association with asthma risk in single-locus analysis performed in our previous study (*P* = 0.21*df* = 2) [19]. A strong synergism or antagonism in the interaction between the ADE genes in determining different types of asthma may suggest that the gene-gene effect can be driven by their true interaction, rather than by the main effect from the distinct gene. These findings may indicate epistatic interactions of the ADE genes, a situation when the effect of one gene may not be disclosed if the effect of another gene is not considered [64].

A post hoc comparative analysis of the frequencies of genotype combinations in the study groups revealed two-locus combinations of the ADE genotypes which increase the risk of the development of asthma. We found relatively rare combinations of genotypes which gave the highest asthma risk estimates but were limited to small subgroups of subjects. In particular, frequencies of these genotype combinations varied from 1 to 9% among healthy controls and from 17 to 41% among patients with nonallergic asthma, whereas odds ratios for disease risk varied from 6.7 to 15.6. Moreover, there was an obvious excess of combinations of variant genotypes among asthma patients compared with healthy subjects, and these differences reached statistical significance after adjusting for multiple tests.

### 4.3. Genetic Variation in ADE Genes and Asthma Pathogenesis

Despite nonsignificant differences in the genotype distributions, the two-locus comparison of genotype frequencies between the study groups has shown that asthma patients more often than healthy subjects carry combinations of the genotypes which are known to determine a diminished activity of ADE towards ROS. This is supported by a number biochemical studies that observed massive generation of ROS and the insufficiency in antioxidant capacity in asthma [65–68]. In this context, it is important to highlight that the studied ADE genes alone cannot account for the whole polygenic mechanisms underlying such biochemical abnormalities in asthma. The interactions between ADE genes that we have identified using different statistical methods make a mechanistic sense because these genes are collectively involved in the maintaining and regulation of redox homeostasis. Moreover, the integrated function of ADE genes in the lung and airways can promote a coordinated detoxification of xenobiotics-induced ROS, thus preventing oxidative stress which plays an important role in the pathogenesis of asthma [21, 24, 51, 69]. Importantly, ADE genes showed interactions with other asthma-related genes such as* IL5* and* IL1B* which are responsible for the immunological mechanisms of asthma and allergy.

Based on the literature data demonstrating biochemical abnormalities in redox homeostasis in asthma and the results of our study, we assumed possible relationships between these abnormalities and ADE genes showed the associations with asthma in our study (the data are shown in Table 7). Changes in the activity of antioxidant defense enzymes such as glutathione peroxidases and catalase in whole blood, plasma, platelets, and bronchoalveolar lavage fluid have been reported by a number of biochemical studies, the findings which are in accordance with our results demonstrating the relationship between the genes for these enzymes and the risk of different pathogenetic variants of asthma. Briefly, an enhanced production of ROS by blood neutrophils, monocytes, and eosinophils found in asthma can be explained by the effects of functional polymorphisms in the gene encoding p22 phox subunit (*CYBA*) of NADPH oxidase. Genetic variation in the* GSR* and* GCLM* genes may be responsible for biochemical perturbations of glutathione metabolism such as an increased level of oxidized glutathione in asthma. Polymorphisms of the* EPHX1* gene determine the increased activity of the enzyme, thus leading to the enhanced production of reactive semiquinones.

Although we did not perform biochemical investigations of antioxidant status, taking the observed association of asthma with the ADE genotypes and their functional significance into account, it is likely that an imbalance between oxidants and antioxidants detected in asthma can be directly related to genetically diminished capacity of ADE. Such an imbalance results in oxidative stress caused by an excessive production of ROS and/or by inadequate antioxidant defense leading to damage of airway epithelial cells and inflammation due to upregulation of redox-sensitive transcription factors and proinflammatory genes [22, 24]. We may also conclude that ADE genes seem to play a greater role in the development of nonallergic asthma than in allergic asthma.

### 4.4. Gender-Specific Effects of ADE Genes on Susceptibility to Bronchial Asthma

An important finding of our study was that polymorphisms of many ADE genes showed sex-specific associations with the development of asthma. For instance, the* CAT* −21A>T and* GCLM* −588C>T gene polymorphisms were associated with asthma susceptibility exclusively in men. In contrast, polymorphism 640A>G of the* CYBA* gene showed a relationship with asthma risk only in women. These findings demonstrate sexual dimorphism in genetic susceptibility to asthma, a phenomenon established for many complex human diseases [70, 71] including asthma [72]. It has been proposed that existing variation in regulatory elements of genes rather than differences in their structure in men and women may explain sex-specific genotype-phenotype interactions in complex traits [70, 73]. Sex-specific changes in age-related gene regulation can result in the difference in asthma susceptibility between the sexes [70]. We suggest that the mechanisms underlying gender-related specificity in the associations of ADE genes with BA found in our study are related to differential expression of redox-sensitive genes in men and women. Since estrogen was found to depress oxidative stress in mice [74], sex-steroid receptors might be an example of sex-specific* trans*-regulatory elements [75] for redox-sensitive genes which in turn may differentially respond to the inducers due to their functionally unequal polymorphic alleles. This means that ADE genes can function differently in men and women in some circumstances. This suggestion is supported by the finding of gender difference in both expression and activity of antioxidant enzymes demonstrated in animal studies [76, 77]. Therefore, it can be concluded that identification of gender-specific genetic variants of ADE, which contribute to the shift of redox homeostasis towards oxidative stress, will provide a better understanding of sex-specific regulation of ADE gene expression and differences in the molecular mechanisms of asthma in men and women.

### 4.5. Limitations of the Study

The study has limitations. Due to the relatively small sample sizes of the studied groups, the association analysis of two-locus genotype combinations was underpowered, especially after Bonferroni adjustment for multiple tests. Because of the limited sample size, we also cannot exclude the possibility that small effects of some ADE genes were not detected. Since BA is a multifactorial and genetically heterogeneous disease [78, 79], further studies with larger sample sizes with genotyping of more polymorphic variants of ADE genes are required for better understanding of the roles of these genes in asthma pathogenesis. Because we did not analyze expression profiles of the genes and biochemical parameters of redox homeostasis, both functional genomics and metabolomics studies are required to clarify the molecular mechanisms by which polymorphisms of ADE genes contribute to the development of BA. Since the risk of BA is determined by a complex interplay between genetic and environmental factors, further genetic studies should take into account environmental factors that may play a significant role in the etiology of the disease.

## 5. Conclusions

To the best of our knowledge, this is the first study investigating the associations between BA and 34 functionally significant polymorphic variants of ADE genes and 12 other candidate genes. So far, no genetic studies have reported a comprehensive evaluation of asthma susceptibility with a number of ADE genes at once. Methodological approaches used in this study were proved fruitful in uncovering the genetic architecture of complex interactions between genes involved in the regulation of redox homeostasis. This allowed finding for the first time that antioxidant defense enzymes genes are collectively involved in the molecular mechanisms of BA and can explain genetic heterogeneity between allergic and nonallergic variants of the disease. In particular, we found for the first time that the* GSR *and* PON2* genes can be referred to as novel asthma susceptibility genes, but their associations need to be confirmed in independent populations. We also showed both complexity and diversity of gene-gene interactions in allergic and nonallergic asthma. Finally, we have discovered gender-specific effects of ADE genes for the risk of the pathogenetic variants of asthma. Altogether the study results provide strong evidence for the pathogenetic role of ADE genes in asthma. Our data on the relationship of the ADE genes and asthma are concordant with the results of a number of biochemical studies demonstrating the massive generation of ROS and the insufficiency in antioxidant capacity which have been implicated in pathogenesis of asthma.

Further studies focusing on the molecular mechanisms regulating redox homeostasis can provide more complete understanding of the role of the ADE genes in bronchial asthma and end up in the discovery of new drug targets for antioxidant treatment and prevention of the disease.

## Supplementary Material

On comparative expression profiles of 23 ADE genes in various cell types/tissues/organs in human

## Figures and Tables

**Table 1:** Description of the polymorphisms included in this study.

Gene symbols (HGNC)		Gene name	Polymorphism (SNP)	Location	SNP ID
1	2	3	4	5	6

1	*GPX1 *	Glutathione peroxidase 1	C>T (P198L)	exon 1	rs1050450
2	*GPX2 *	Glutathione peroxidase 2 (gastrointestinal)	G>A (R146C)	exon 2	rs17880492
3	*GPX3 *	Glutathione peroxidase 3 (plasma)	249G>A	3′ UTR	rs2070593
4	*GPX4 *	Glutathione peroxidase 4 (phospholipid hydroperoxidase)	C718T	3′ UTR	rs713041
5	*GSR *	Glutathione reductase	T>C (30546636T>C)	intron 9	rs2551715
6	*SOD2 *	Superoxide dismutase 2, mitochondrial	A16V	exon 2	rs4880
7	*SOD3 *	Superoxide dismutase 3, extracellular	A40T (A58T)	exon 3	rs2536512
8	*CAT *	Catalase —//—	−21A>T (−89A>T)	5′ UTR	rs7943316
9	*CAT *		−262C>T (4760C>T)	5′ UTR	rs1001179
10	*GCLM *	Glutamate-cysteine ligase, modifier subunit	−588C>T (4704C>T)	5′ UTR	rs41303970
11	*GCLM *		−23G>T	5′UTR	rs743119
12	*NQO1 *	NAD(P)H dehydrogenase, quinone 1 —//—	P187S	exon 6	rs1800566
13	*NQO1 *		R139W	exon 4	rs4986998
14	*CYBA *	Cytochrome b-245, alpha polypeptide —//— —//—	242C>T (Y72H)	exon 4	rs4673
15	*CYBA *		640A>G (24G>A)	3′ UTR	rs1049255
16	*CYBA *		−930A>G	5′ UTR	rs9932581
17	*MPO *	Myeloperoxidase	−463G>A (4535G>A)	5′ UTR	rs2333227
18	*PRDX1 *	Peroxiredoxin 1	C>A	5′ UTR	rs17522918
19	*TXNRD1 *	Thioredoxin reductase 1	C>G	5′ UTR	rs1128446
20	*FMO3 *	Flavin-containing monooxygenase 3	E158K	exon 4	rs2266782
21	*CYP1A1 *	Cytochrome P450, family 1, subfamily A, polypeptide 1	I462V	exon 7	rs1048943
22	*CYP1A1 *		T6235C	3′ UTR	rs4646903
23	*CYP2E1 *	Cytochrome P450, family 2, subfamily E, polypeptide 1 —//— —//—	−1293G>C	5′ UTR	rs3813867
24	*CYP2E1 *		−1053C>T	5′ UTR	rs2031920
25	*CYP2E1 *		7632T>A	intron 6	rs6413432
26	*CYP2E1 *		9896C>G	intron 7	rs2070676
27	*EPHX1 *	Epoxide hydrolase 1, microsomal (xenobiotic)	Y113H (337T>C)	exon 3	rs1051740
28	*EPHX1 *		H139R (416A>G)	exon 4	rs2234922
29	*PON1 *	Paraoxonase 1	Q192R	exon 6	rs662
30	*PON2 *	Paraoxonase 2	C311S	exon 9	rs7493
31	*GSTM1 *	Glutathione S-transferase mu 1	Expressor/deletion	exons 6-7	—
32	*GSTT1 *	Glutathione S-transferase theta 1	Expressor/deletion	exon 4	—
33	*GSTP1 *	Glutathione S-transferase pi 1 —//—	I105V	exon 5	rs1695
34	*GSTP1 *		A114V	exon 6	rs1138272
35	*TNF *	Tumor necrosis factor	−308G>A	5′ UTR	rs1800629
36	*IL1B *	Interleukin 1, beta	−511C>T	5′ UTR	rs16944
37	*IL3 *	Interleukin 3 (colony-stimulating factor, multiple)	S27P	exon 1	rs40401
38	*IL3 *		−15C>T	5′ UTR	rs31480
39	*IL5 *	Interleukin 5 (colony-stimulating factor, eosinophil)	C-703T	5′ UTR	rs2069812
40	*CSF2RB (IL5RB) *	Colony stimulating factor 2 receptor, beta, low-affinity (granulocyte-macrophage)	G1972A	exon 5	rs131840
41	*IL9 *	Interleukin 9	T113M	exon 5	rs2069885
42	*IL13 *	Interleukin 13	−1111C>T	5′ UTR	rs1800925
43	*SCGB1A1 (CC16) *	Secretoglobin, family 1A, member 1 (uteroglobin)	A38G	exon 1	rs11549442
44	*SERPINA1 *	Serpin peptidase inhibitor, clade A (alpha-1 antiproteinase, antitrypsin), member 1	E288V	exon 3	rs17580
45	*SERPINA1 *		D365N	exon 5	rs143370956
46	*SERPINA1 *		1331G>A	3′ UTR	rs11568814

**Table 2:** Allele frequencies of genes investigated in the present study.

Gene	Polymorphism	Alleles	Allele frequency			
			Controls (*n* = 214)	Asthma, entire group (*n* = 215)	Allergic asthma (*n* = 156)	Nonallergic asthma (*n* = 56)
1	2	3	4	5	6	7

*GPX2 *	G>A (rs17880492)	G	0.991	0.981	0.987	0.964
		A	0.009	0.019	0.013	0.036
*GPX3 *	G>A (rs2070593)	G	0.703	0.726	0.734	0.696
		A	0.297	0.274	0.266	0.304
*GPX4 *	C718T (rs713041)	718T	0.402	0.391	0.407	0.348
		718C	0.598	0.609	0.593	0.652
*GSR *	T>C (rs2551715)	T	0.442	0.398	0.362	0.491
		C	0.558	0.602	0.638*	0.509
*SOD2 *	A16V (rs4880)	16A	0.528	0.486	0.481	0.509
		16V	0.472	0.514	0.519	0.491
*SOD3 *	A40T (rs2536512)	40A	0.322	0.321	0.324	0.295
		40T	0.678	0.679	0.676	0.705
*PRDX1 *	C>A (rs17522918)	C	0.923	0.937	0.942	0.920
		A	0.077	0.063	0.058	0.080
*TXNRD1 *	C>G (rs1128446)	C	0.808	0.821	0.808	0.857
		G	0.192	0.179	0.192	0.143
*FMO3 *	E158K (rs2266782)	158E	0.549	0.537	0.529	0.571
		158K	0.451	0.463	0.471	0.429
*TNF *	−308G>A (rs1800629)	−308G	0.888	0.872	0.875	0.866
		−308A	0.112	0.128	0.125	0.134
*IL1B *	−511C>T (rs16944)	−511C	0.710	0.664	0.670	0.652
		−511T	0.290	0.336	0.330	0.348
*IL3 *	S27P (rs40401)	27S	0.738	0.685	0.686	0.688
		27P	0.262	0.315	0.314	0.313
*IL3 *	−15C>T (rs31480)	−15C	0.741	0.683	0.686	0.688
		−15T	0.259	0.317	0.314	0.313
*IL5 *	C-703T (rs2069812)	−703C	0.673	0.778	0.788	0.732
		−703T	0.327	0.222*	0.212*	0.268
*IL5RB (CSF2RB) *	G1972A (rs131840)	1972G	0.831	0.866	0.872	0.848
		1972A	0.169	0.134	0.128	0.152
*IL9 *	T113M (rs2069885)	113T	0.820	0.863	0.856	0.893
		113M	0.180	0.137	0.144	0.107
*IL13 *	−1111C>T (rs1800925)	−1111C	0.729	0.693	0.692	0.714
		−1111T	0.271	0.307	0.308	0.286
*CC16 (SCGB1A1) *	A38G (rs11549442)	38A	0.347	0.367	0.372	0.366
		38G	0.653	0.633	0.628	0.634
*SERPINA1 *	E288V (rs17580)	288E	0.993	0.991	0.990	0.991
		288V	0.007	0.009	0.010	0.009
*SERPINA1 *	D365N (rs143370956)	365D	0.991	0.995	0.997	0.991
		365N	0.009	0.005	0.003	0.009
*SERPINA1 *	1331G>A (rs11568814)	1331G	0.937	0.933	0.926	0.946
		1331A	0.063	0.067	0.074	0.054

*Indicates  a difference in minor allele frequency between asthmatics and controls.

**Table 3:** Genotype frequencies of genes investigated in the present study.

Gene	Polymorphism	Genotypes	Genotype distributions, *n* (%)							
			Controls (*n* = 214)		Asthma, entire group (*n* = 215)		Allergic asthma (*n* = 156)		Nonallergic asthma (*n* = 56)	
1	2	3	4	5	6	7	8	9	10	11

*GPX2 *	G>A (rs17880492)	GG	210	98.1	207	96.3	152	97.4	52	92.9
		GA	4	1.9	8	3.7	4	2.6	4	7.1
		AA	0	0.0	0	0.0	0	0.0	0	0.0
*GPX3 *	G>A (rs2070593)	GG	105	49.1	113	52.6	83	53.2	28	50.0
		GA	91	42.5	86	40.0	63	40.4	22	39.3
		AA	18	8.4	16	7.4	10	6.4	6	10.7
*GPX4 *	C718T (rs713041)	718TT	31	14.5	33	15.3	25	16.0	8	14.3
		718TC	110	51.4	102	47.4	77	49.4	23	41.1
		718CC	73	34.1	80	37.2	54	34.6	25	44.6
*GSR *	T>C (rs2551715)	TT	40	18.7	32	14.9	17	10.9*	15	26.8
		TC	109	50.9	107	49.8	79	50.6	25	44.6
		CC	65	30.4	76	35.3	60	38.5	16	28.6
*SOD2 *	A16V (rs4880)	16AA	59	27.6	49	22.8	34	21.8	15	26.8
		16AV	108	50.5	111	51.6	82	52.6	27	48.2
		16VV	47	22.0	55	25.6	40	25.6	14	25.0
*SOD3 *	A40T (rs2536512)	40AA	21	9.8	24	11.2	19	12.2	4	7.1
		40AT	96	44.9	90	41.9	63	40.4	25	44.6
		40TT	97	45.3	101	47.0	74	47.4	27	48.2
*PRDX1 *	C>A (rs17522918)	CC	182	85.0	188	87.4	138	88.5	47	83.9
		CA	31	14.5	27	12.6	18	11.5	9	16.1
		AA	1	0.5	0	0.0	0	0.0	0	0.0
*TXNRD1 *	C>G (rs1128446)	CC	140	65.4	145	67.4	101	64.7	42	75.0
		CG	66	30.8	63	29.3	50	32.1	12	21.4
		GG	8	3.7	7	3.3	5	3.2	2	3.6
*FMO3 *	E158K (rs2266782)	158EE	57	26.6	59	27.4	39	25.0	20	35.7
		158EK	121	56.5	113	52.6	87	55.8	24	42.9
		158KK	36	16.8	43	20.0	30	19.2	12	21.4
*TNF *	−308G>A (rs1800629)	−308GG	170	79.4	162	75.4	118	75.6	42	75.0
		−308GA	40	18.7	51	23.7	37	23.7	13	23.2
		−308AA	4	1.9	2	0.9	1	0.6	1	1.8
*IL1B *	−511C>T (rs16944)	−511CC	114	53.3	91	42.1	67	43.0*	23	41.1
		−511CT	76	35.5	105	48.6	75	48.1*	27	48.2
		−511TT	24	11.2	20	9.3	14	9.0	6	10.7
*IL3 *	S27P (rs40401)	27SS	120	56.1	104	48.2	77	49.4	25	44.6
		27SP	76	35.5	88	40.7	60	38.5	27	48.2
		273P	18	8.4	24	11.1	19	12.2	4	7.1
*IL3 *	−15C>T (rs31480)	−15CC	120	56.1	103	47.7	77	49.4	25	44.6
		−15CT	77	36.0	89	41.2	60	38.5	27	48.2
		−15TT	17	7.9	24	11.1	19	12.2	4	7.1
*IL5 *	C-703T (rs2069812)	−703CC	90	42.1	132	61.1	97	62.2*	31	55.4
		−703CT	108	50.5	72	33.3	52	33.3*	20	35.7*
		−703TT	16	7.5	12	5.6	7	4.5	5	8.9
*IL5RB* *(CSF2RB) *	G1972A (rs131840)	1972GG	136	67.7	160	74.1	118	75.6	39	69.6
		1972GA	62	30.9	54	25.0	36	23.1	17	30.4
		1972AA	3	1.5	2	0.9	2	1.3	0	0.0
*IL9 *	T113M (rs2069885)	113TT	146	68.2	159	73.6	113	72.4	44	78.6
		113TM	59	27.6	55	25.5	41	26.3	12	21.4
		113MM	9	4.2	2	0.9	2	1.3	0	0.0
*IL13 *	−1111C>T (rs1800925)	−1111CC	114	53.3	101	47.0	75	48.1	26	46.4
		−1111CT	84	39.3	96	44.7	66	42.3	28	50.0
		−1111CT	16	7.5	18	8.4	15	9.6	2	3.6
*CC16* *(SCGB1A1) *	A38G (rs11549442)	38AA	25	11.8	28	13.0	23	14.7	5	8.9
		38AG	97	45.8	102	47.4	70	44.9	31	55.4
		38GG	90	42.5	85	39.5	63	40.4	20	35.7
*SERPINA1 *	E288V (rs17580)	288EE	211	98.6	212	98.1	153	98.1	55	98.2
		288EV	3	1.4	4	1.9	3	1.9	1	1.8
		288VV	—	—	—	—	—	—	—	—
*SERPINA1 *	D365N (rs143370956)	365DD	210	98.1	214	99.1	153	98.1	55	98.2
		365DN	4	1.9	2	0.9	3	1.9	1	1.8
		365NN	—	—	—	—	—	—	—	—
*SERPINA1 *	1331G>A (rs11568814)	1331GG	188	87.9	187	86.6	133	85.3	50	89.3
		1331GA	25	11.7	29	13.4	23	14.7	6	10.7
		1331AA	1	0.5	0	0.0	0	0.0	0	0.0

*Indicates  a difference in genotype frequency between asthmatics and controls.

**Table 4:** A summary of best 2-, 3-, and 4-locus models of gene-gene interactions obtained by MDR analysis in allergic and nonallergic asthma (exhaustive search algorithm).

Number of loci	Best *n*-locus (2-, 3-, and 4-locus) models of gene-gene interactions	Cross-validation consistency, %	Prediction error, %
Allergic asthma (men)			
2	*CYP2E1 *9896C>G × *IL5* C-703T	40	52.5
3	*CAT* −21A>T × *IL5* C-703T × *GSR* T/C*	50	50.3
4	*CAT* −21A>T × *GPX1 *P198L × *PON2* S311C × *IL3* S27P	30	53.6

Allergic asthma (women)			
2	*EPHX1* Y113H × *IL5* C-703T	50	45.8
3	*EPHX1* Y113H × *IL5* C-703T × *GPX1 *P198L**	50	40.9
4	*EPHX1* Y113H × *CYBA* 640A>G × *GPX4 *C718T × *PON2* S311C	20	50.1

Nonallergic asthma (men)			
2	*GPX3 *G/A × *GCLM *−588C>T	30	52.3
3	*GCLM *−588C>T × *GSR* T/C × *CYBA* 242C>T	20	57.0
4	*GPX3 *G/A × *FMO3 *E158K × *GPX1 *P198L × *CYBA* −930A>G*	30	49.2

Nonallergic asthma (women)			
2	*EPHX1* Y113H × *IL3* S27P	30	46.7
3	*EPHX1* Y113H × *GSR* T/C × *SOD2 *A16V	60	45.5
4	*EPHX1* Y113H × *GSR* T/C × *SOD2 *A16V × *CYBA* 640A>G*	90	27.4

*Indicates  best *n*-locus model of gene-gene interactions evaluated through 1000 permutation tests.

**A statistically significant (*P* value 0.001) model of gene-gene interactions.

**Table 5:** Associations of genotype combinations with risk of allergic asthma (stratified by gender).

Combinations of genotypes	Allergic asthma		Controls		Chi-square (*P* value^1^)	OR (95% CI)
	*N*	%	*N*	%		
Men						
*GPX1 *198PL × *GPX2 *GG	34	53.1	38	36.2	4.66 (0.03)	2.00 (1.06–3.76)
*GPX1 *198PL × *GSR* TC	20	31.3	19	18.1	3.88 (0.05)	2.06 (1.00–4.25)
*GPX1 *198PL × *CAT* −21AA	7	10.9	2	1.9	4.77 (0.03)	5.40 (1.25–23.42)
*GPX1 *198PL × *GCLM* −588CT	12	18.8	6	5.7	5.80 (0.02)	3.64 (1.33–9.96)
*GPX1 *198PL ×* IL5* −703CC	18	28.1	13	12.4	6.58 (0.01)	2.77 (1.25–6.14)
*GPX2 *GG × *CAT* −21AA	12	18.8	7	6.7	4.67 (0.03)	3.13 (1.19–8.21)
*GPX2 *GG ×* IL5* −703CC	38	59.4	44	41.9	4.86 (0.03)	2.03 (1.08–3.81)
*GSR* CC × *IL5* −703CC	17	26.6	11	10.5	7.44 (0.01)	3.09 (1.34–7.13)
*CAT* −21AA ×* IL5* −703CC	9	14.1	3	2.9	5.97 (0.01)	5.01 (1.41–17.8)
*CAT* −21AT ×* IL5* −703CT	6	9.4	28	26.7	6.36 (0.01)	0.30 (0.12–0.76)

Women						
*NQO1* 187PP × *IL5* −703CC	43	46.7	33	30.3	5.75 (0.02)	2.02 (1.13–3.60)
*NQO1 *187PP × *IL5* −703CT	18	19.6	39	35.8	6.46 (0.01)	0.44 (0.23–0.83)
*NQO1 *187PP × *IL5* −703CC	43	46.7	33	30.3	5.75 (0.02)	2.02 (1.13–3.60)
*NQO1 *187PP × *IL5* −703CT	18	19.6	39	35.8	6.46 (0.01)	0.44 (0.23–0.83)
*EPHX1 *113YY × *IL5* −703CT	9	9.8	24	22.0	4.59 (0.03)	0.40 (0.18–0.89)
*EPHX1 *113YY × *IL5* −703CT	9	9.8	24	22.0	4.59 (0.03)	0.40 (0.18–0.89)
*GPX1 *198PL × *GPX4 *718TT	11	12.0	4	3.7	3.83 (0.05)	3.31 (1.07–10.22)
*GPX1 *198PP × *CYBA* 640AG	13	14.1	31	28.4	5.97 (0.01)	0.41 (0.20–0.85)
*GPX4 *718TC × *CYBA* 640AG	18	19.6	43	39.4	9.33 (0.002)*	0.37 (0.20–0.71)

^1^Means  unadjusted *P* value. *P* value of 0.002 (*P*_adj_: adjusted for multiple tests) was set as statistically significant (*a statistically significant association).

**Table 6:** Associations of genotype combinations with risk of nonallergic asthma (stratified by gender).

Combinations of genotypes	Nonallergic asthma		Controls		Chi-square (*P* value^1^)	OR (95% CI)
	*N*	%	*N*	%		
Men						
*GPX1 *198PL × *CAT* −21AA	6	20.7	2	1.9	11.13 (0.001)*	11.45 (2.49–52.66)
*GPX1 *198LL × *GCLM* −588CT	4	13.8	3	2.9	3.50 (0.05)	5.17 (1.20–22.31)
*GPX3 *GA × *FMO3 *158KK	5	17.2	4	3.8	4.58 (0.03)	5.06 (1.37–18.99)
*GPX3 *GA × *GSR* TT	6	20.7	5	4.8	5.68 (0.02)	5.05 (1.49–17.14)
*GPX3 *GA × *CAT* −21AA	5	17.2	4	3.8	4.58 (0.03)	5.06 (1.35–18.99)
*GPX3 *GG × *GCLM* −588CT	11	37.9	15	14.3	8.12 (0.004)	3.67 (1.47–9.28)
*GSR* TT × *GCLM* −588CT	8	27.6	3	2.9	15.31 (0.0001)*	11.58 (3.07–43.72)
*GSR* TT × *FMO3 *158KK	4	13.8	1	1.0	7.16 (0.01)	12.29 (1.84–82.03)
*CAT* −21AA × *CYBA* −930GG	5	17.2	1	1.0	10.55 (0.001)*	15.64 (2.44–100.3)
*CAT* −21AA × *FMO3 *158EE	4	13.8	1	1.0	7.16 (0.01)	12.29 (1.84–82.03)
*GCLM* −588CT × *CYBA* −930GG	12	41.4	10	9.5	16.8 (0.00004)*	6.71 (2.5–17.96)
*CYBA* −930GG × *FMO3 *158EE	7	24.1	5	4.8	8.22 (0.004)	6.09 (1.85–20.05)

Women						
*GPX1 *198PL × *GPX2 *GA	2	7.4	0	0.0	3.88 (0.05)	21.47 (1.00–461.1)
*GPX2 *GG ×* IL5* −703CT	6	22.2	56	51.4	6.29 (0.01)	0.29 (0.11–0.74)
*GPX2 *GA × *IL5* −703CC	2	7.4	0	0.0	3.88 (0.05)	21.5 (1.00–461.2)
*EPHX1 *113YH × *IL5* −703CT	1	3.7	24	22.0	3.69 (0.05)	0.20 (0.04–1.09)
*EPHX1 *113HH × *IL5* −703CC	7	25.9	4	3.7	11.58 (0.001)*	8.58 (2.43–30.26)

^1^Means  unadjusted  *P* value. *P* value adjusted for multiple tests (*P*_adj_) is less than 0.002 in men and 0.004 in women. *A statistically significant association.

**Table 7:** Common biochemical abnormalities in redox homeostasis found in asthma and their possible relationship with genes for antioxidant defense enzymes which have been associated with risk of the disease in the present study.

Biochemical abnormalities in asthmatics [references]	ADE gene related with the abnormality	Allergic asthma		Nonallergic asthma	
		Men	Women	Men	Women
Diminished capacity of glutathione peroxidases and catalase in detoxification of hydrogen peroxide [22, 38–43].	*GPX1 *	+ + +	+ +	+ +	+ +
	*GPX2 *	+ +	−		+ + +
	*GPX3 *	−	−	+ +	−
	*GPX4 *	−	+	−	−
	*CAT *	+ + +	−	+ +	−

An enhanced production of ROS/hydrogen peroxide/superoxide anion radicals [22, 44–50].	*CYBA* (640A>G)	−	+	−	−
	*CYBA* (−930A>G)	−	−	+ + +	−

Perturbations in glutathione (GSH) homeostasis [41, 48, 50–52].	*GSR *	+ +	−	+ + +	−
	*GCLM *	+ +	−	+ + +	−

Increased EPHX1 activity, increased production of xenobiotics-generated epoxides, *trans*-dihydrodiols and reactive semiquinones resulting in ROS generation [53, 54].	*EPHX1 *	+ +	+ + +	+ +	+ + +

The number of pluses means a degree of the relationship between the gene and asthma risk. These measures reflect how many times a particular gene showed the link with asthma risk through the three methods used for evaluation of gene-gene interactions in the present study, namely, set association approach (SAA), multifactor dimensionality reduction (MDR) method, and post hoc association analysis of two-locus genotype combinations (AAGC): + + + means that the link was found thrice (i.e., using SAA, MDR, and AAGC methods); + + means that the link was found twice (i.e., using SAA or MDR and AAGC methods); + means that the link was found once by AAGC method. Associations are stratified by asthma type and gender.
