# Supplementary material for: Antioxidant Defense Enzyme Genes and Asthma Susceptibility: Gender-Specific Effects and Heterogeneity in Gene-Gene Interactions between Pathogenetic Variants of the Disease
Source: Biomed Res Int. 2014 May 5;2014:708903. doi: 10.1155/2014/708903 (PMC4026955; doi:10.1155/2014/708903)
Supplement: Supplementary file 1 — On comparative expression profiles of 23 ADE genes in various cell types/tissues/organs in human [file 708903.f1.pdf]

Supplementary material

Comparative expression profiles of genes for antioxidant defense enzymes in various cell types/tissues/organs in human

(Data retrieved from BioGPS web site, http://biogps.org)*

GPX1

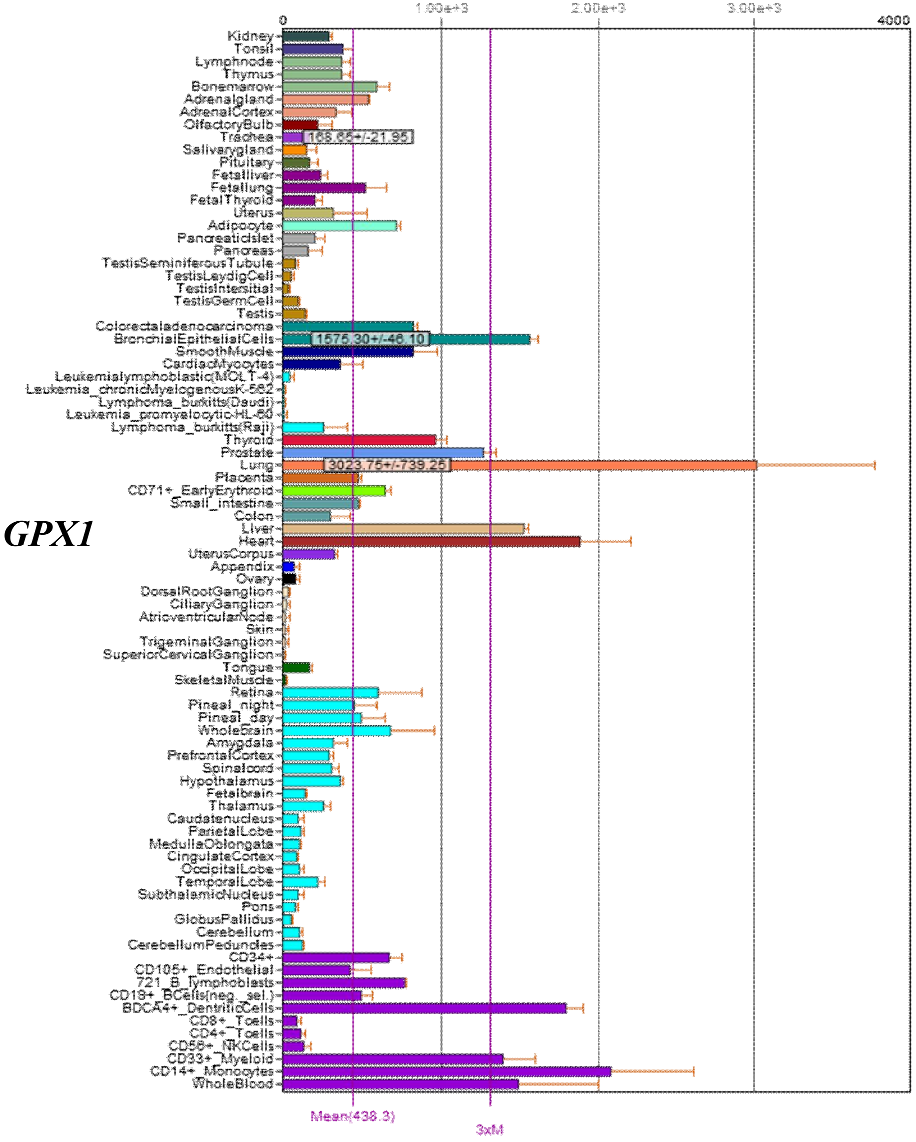

GPX2

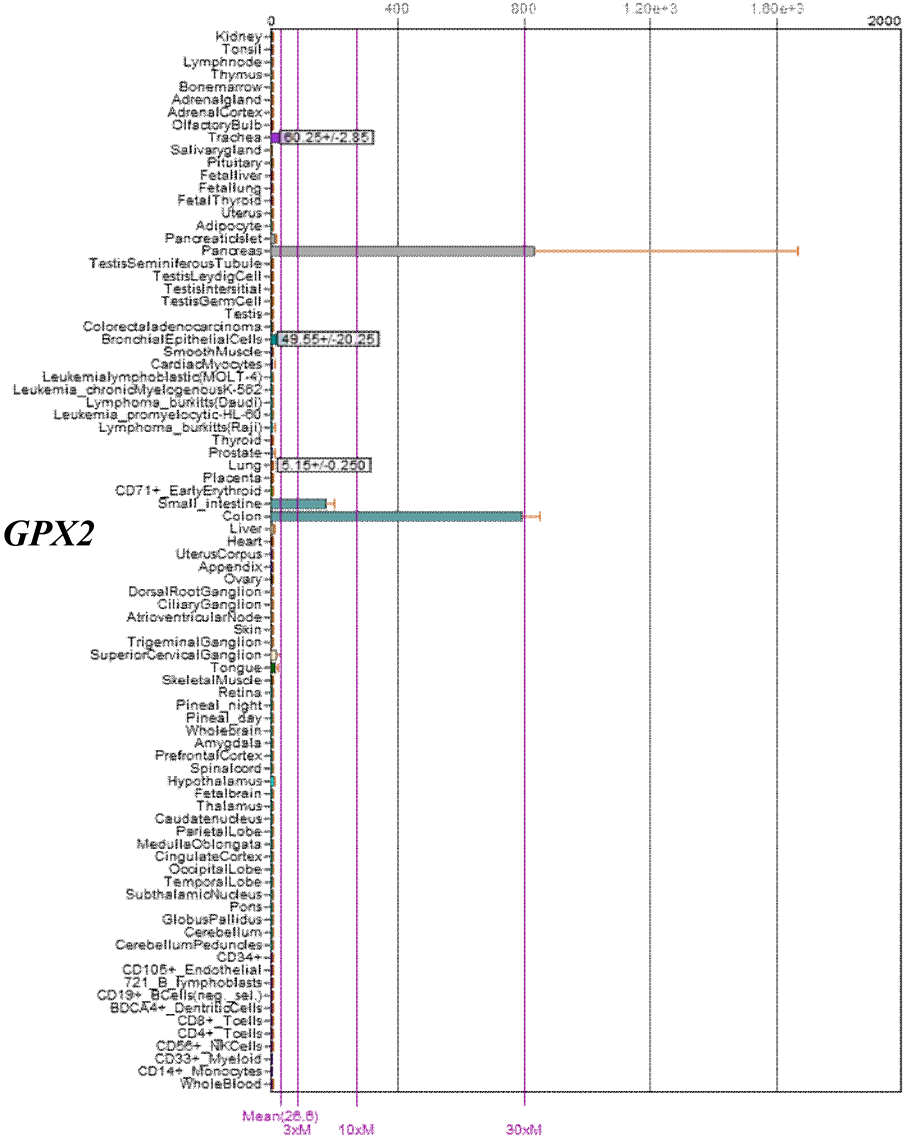

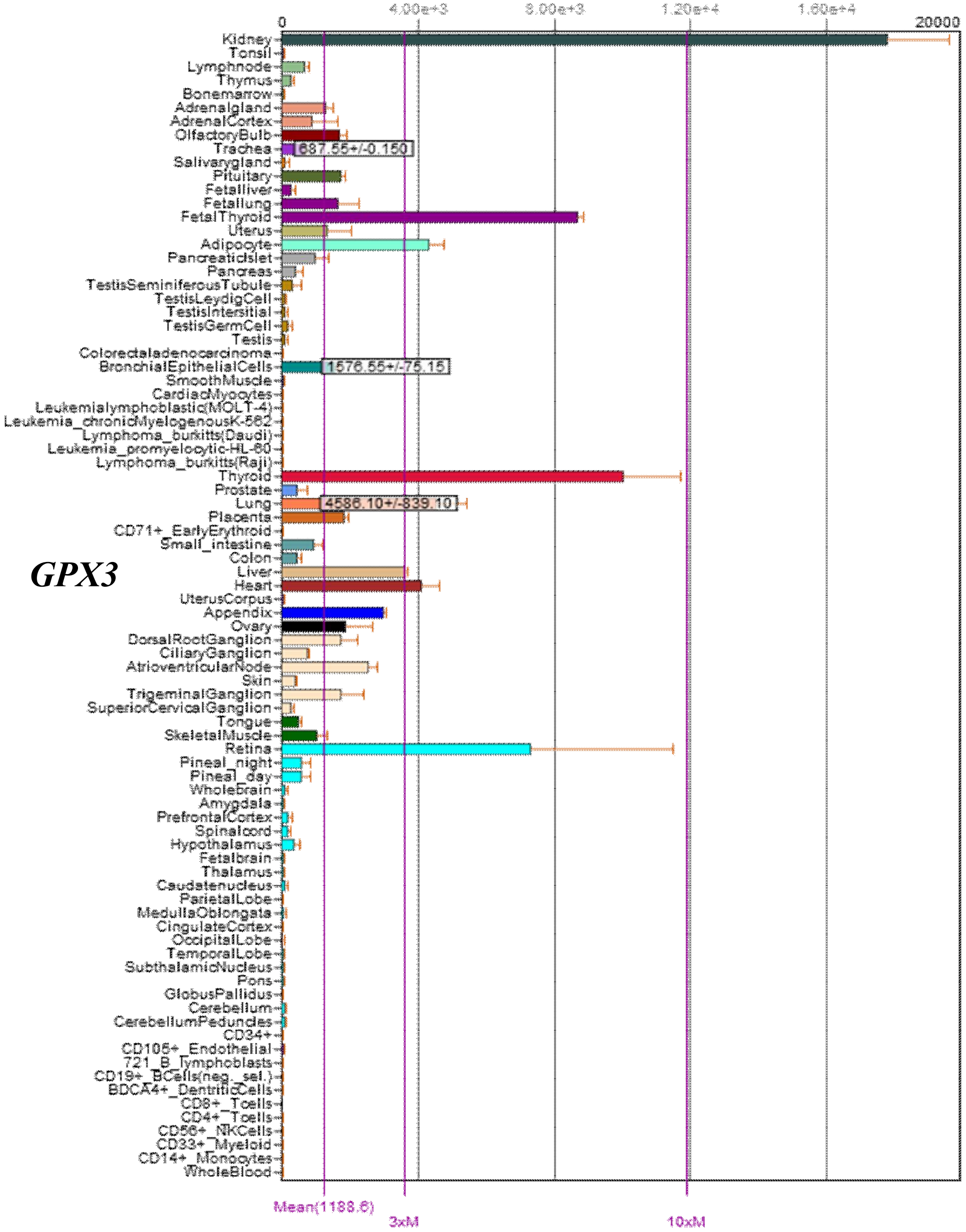
0
4.00e+3
8.00e+3
1.20e+4
1.60e+4
20000
Kidney
Tonsil
Lymphnode
Thymus
Bonemarrow
Adrenalgland
AdrenalCortex
OlfactoryBulb
Trachea
687.55+/-0.150
Salivarygland
Pituitary
Fetalliver
Fetallung
FetalThyroid
Uterus
Adipocyte
Pancreaticlslet
Pancreas
TestisSeminiferousTubule
TestisLeydigCell
TestisIntersitial
TestisGermCell
Testis
Colorectaladenocarcinoma
BronchialEpithelialCells
1576.55+/-75.15
SmoothMuscle
CardiacMyocytes
Leukemialymphoblastic(MOLT-4)
Leukemia_chronicMyelogenousK-562
Lymphoma_burkitts(Daudi)
Leukemia_promyelocytic-HL-60
Lymphoma_burkitts(Raji)
Thyroid
Prostate
Lung
4586.10+/-839.10
Placenta
CD71+_EarlyErythroid
Small_intestine
Colon
Liver
Heart
UterusCorpus
Appendix
Ovary
DorsalRootGanglion
CiliaryGanglion
AtrioventricularNode
Skin
TrigeminalGanglion
SuperiorCervicalGanglion
Tongue
SkeletalMuscle
Retina
Pineal_night
Pineal_day
WholeBrain
Amygdala
PrefrontalCortex
Spinalcord
Hypothalamus
Fetalbrain
Thalamus
Caudatenucleus
ParietalLobe
MedullaOblongata
CingulateCortex
OccipitalLobe
TemporalLobe
SubthalamicNucleus
Pons
GlobusPallidus
Cerebellum
CerebellumPeduncles
CD34+
CD105+_Endothelial
721_B_lymphoblasts
CD19+_BCells(neg._sel.)
BDCA4+_DentriticCells
CD8+_Tcells
CD4+_Tcells
CD56+_NKCells
CD33+_Myeloid
CD14+_Monocytes
WholeBlood
Mean(1188.6)
3xM
10xM

GPX3

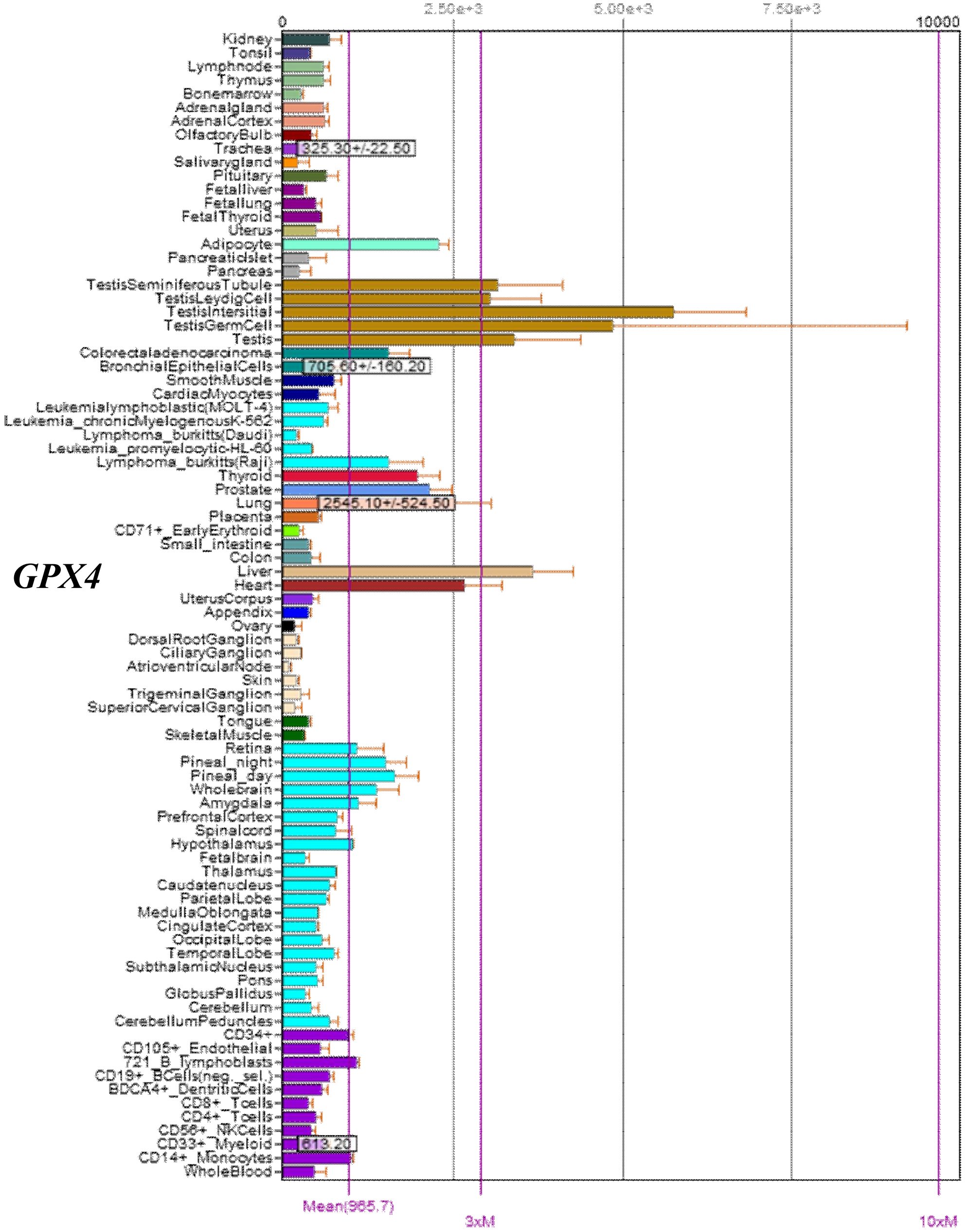
0
2.50e+3
5.00e+3
7.50e+3
10000
Kidney
Tonsil
Lymphnode
Thymus
Bonemarrow
Adrenalgland
AdrenalCortex
OlfactoryBulb
Trachea
325.30+/-22.50
Salivarygland
Pituitary
Fetalliver
Fetallung
FetalThyroid
Uterus
Adipocyte
Pancreaticlslet
Pancreas
TestisSeminiferousTubule
TestisLeydigCell
TestisIntersitial
TestisGermCell
Testis
Colorectaladenocarcinoma
BronchialEpithelialCells
705.60+/-160.20
SmoothMuscle
CardiacMyocytes
Leukemialymphoblastic(MOLT-4)
Leukemia_chronicMyelogenousK-562
Lymphoma_burkitts(Daudi)
Leukemia_promyelocytic-HL-60
Lymphoma_burkitts(Raji)
Thyroid
Prostate
Lung
2545.10+/-524.50
Placenta
CD71+_EarlyErythroid
Small_intestine
Colon
Liver
Heart
UterusCorpus
Appendix
Ovary
DorsalRootGanglion
CiliaryGanglion
AtrioventricularNode
Skin
TrigeminalGanglion
SuperiorCervicalGanglion
Tongue
SkeletalMuscle
Retina
Pineal_night
Pineal_day
WholeBrain
Amygdala
PrefrontalCortex
Spinalcord
Hypothalamus
Fetalbrain
Thalamus
Caudatenucleus
ParietalLobe
MedullaOblongata
CingulateCortex
OccipitalLobe
TemporalLobe
SubthalamicNucleus
Pons
GlobusPallidus
Cerebellum
CerebellumPeduncles
CD34+
CD105+_Endothelial
721_B_lymphoblasts
CD19+_BCells(neg._sel.)
BDCA4+_DentriticCells
CD8+_Tcells
CD4+_Tcells
CD56+_NKCells
CD33+_Myeloid
613.20
CD14+_Monocytes
WholeBlood
Mean(965.7)
3xM
10xM

GPX4

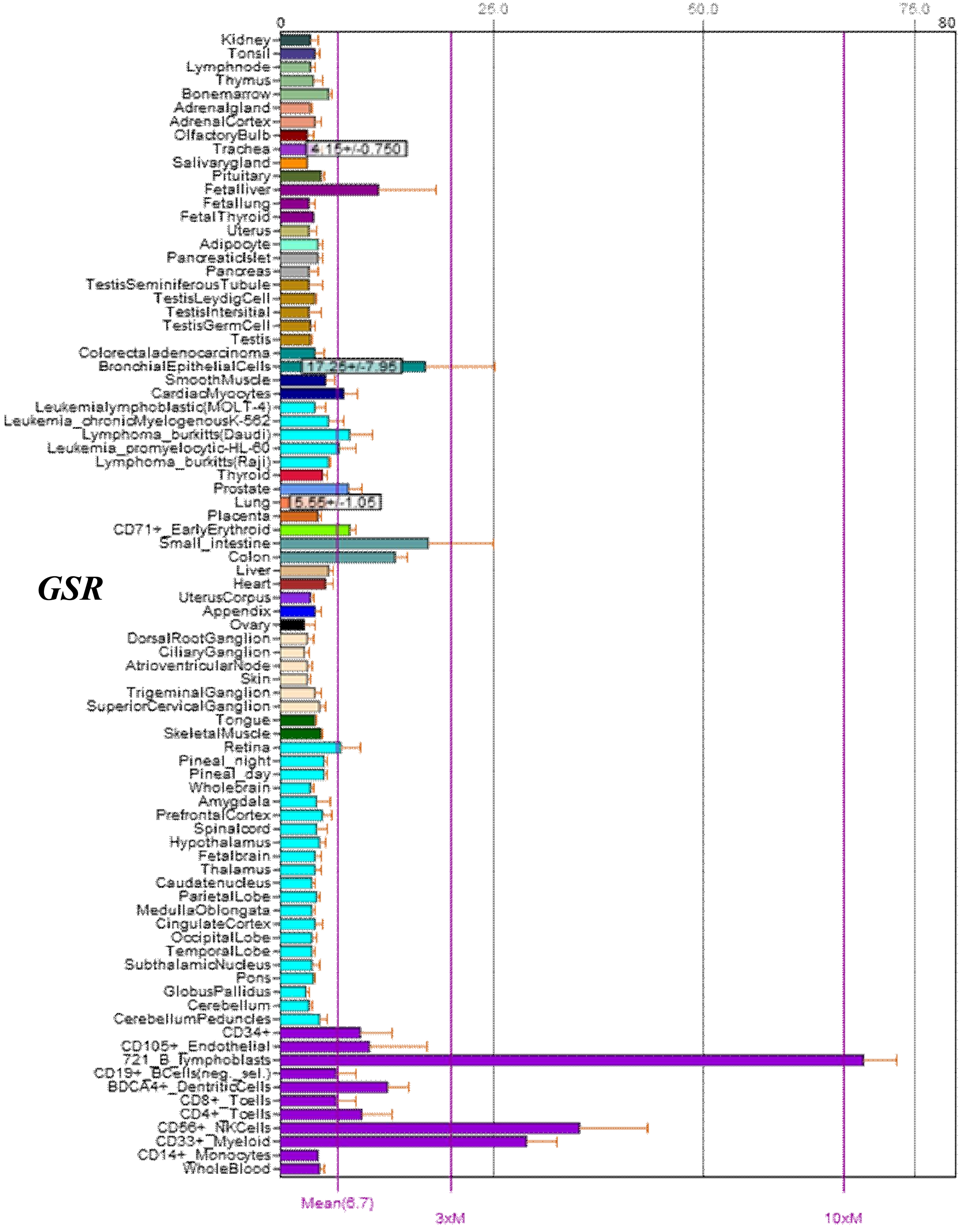
GSR
0
25.0
50.0
75.0
80
Kidney
Tonsil
Lymphnode
Thymus
Bonemarrow
Adrenalgland
AdrenalCortex
OlfactoryBulb
Trachea
4.15+/-0.750
Salivarygland
Pituitary
Fetalliver
Fetallung
FetalThyroid
Uterus
Adipocyte
PancreaticIslet
Pancreas
TestisSeminiferousTubule
TestisLeydigCell
TestisIntersitial
TestisGermCell
Testis
Colorectaladenocarcinoma
BronchialEpithelialCells
17.25+/-7.95
SmoothMuscle
CardiacMyocytes
Leukemialymphoblastic(MOLT-4)
Leukemia_chronicMyelogenousK-562
Lymphoma_burkitts(Daudi)
Leukemia_promyelocytic-HL-60
Lymphoma_burkitts(Raji)
Thyroid
Prostate
Lung
5.55+/-1.05
Placenta
CD71+_EarlyErythroid
Small_intestine
Colon
Liver
Heart
UterusCorpus
Appendix
Ovary
DorsalRootGanglion
CiliaryGanglion
AtrioventricularNode
Skin
TrigeminalGanglion
SuperiorCervicalGanglion
Tongue
SkeletalMuscle
Retina
Pineal_night
Pineal_day
WholeBrain
Amygdala
PrefrontalCortex
Spinalcord
Hypothalamus
Fetalbrain
Thalamus
Caudatenucleus
ParietalLobe
MedullaOblongata
CingulateCortex
OccipitalLobe
TemporalLobe
SubthalamicNucleus
Pons
GlobusPallidus
Cerebellum
CerebellumPeduncles
CD34+
CD105+_Endothelial
721_B_lymphoblasts
CD19+_BCells(neg._sel.)
BDCA4+_DentriticCells
CD8+_Tcells
CD4+_Tcells
CD56+_NKCells
CD33+_Myeloid
CD14+_Monocytes
WholeBlood
Mean(6.7)
3xM
10xM

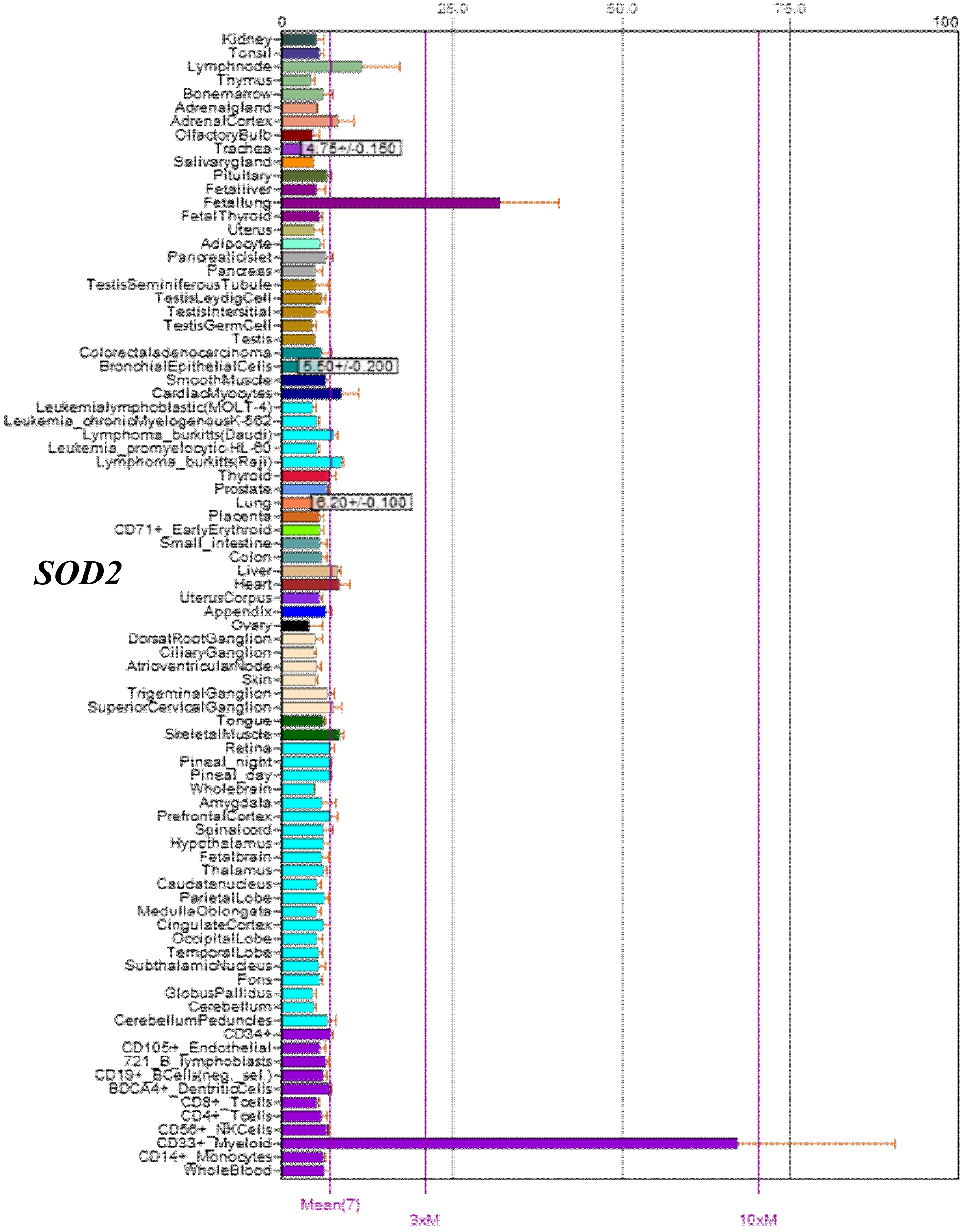
SOD2
0
25.0
50.0
75.0
100
Kidney
Tonsil
Lymphnode
Thymus
Bonemarrow
Adrenalgland
AdrenalCortex
OlfactoryBulb
Trachea
4.75+/-0.150
Salivarygland
Pituitary
Fetalliver
Fetallung
FetalThyroid
Uterus
Adipocyte
PancreaticIslet
Pancreas
TestisSeminiferousTubule
TestisLeydigCell
TestisIntersitial
TestisGermCell
Testis
Colorectaladenocarcinoma
BronchialEpithelialCells
5.50+/-0.200
SmoothMuscle
CardiacMyocytes
Leukemialymphoblastic(MOLT-4)
Leukemia_chronicMyelogenousK-562
Lymphoma_burkitts(Daudi)
Leukemia_promyelocytic-HL-60
Lymphoma_burkitts(Raji)
Thyroid
Prostate
Lung
6.20+/-0.100
Placenta
CD71+_EarlyErythroid
Small_intestine
Colon
Liver
Heart
UterusCorpus
Appendix
Ovary
DorsalRootGanglion
CiliaryGanglion
AtrioventricularNode
Skin
TrigeminalGanglion
SuperiorCervicalGanglion
Tongue
SkeletalMuscle
Retina
Pineal_night
Pineal_day
WholeBrain
Amygdala
PrefrontalCortex
Spinalcord
Hypothalamus
Fetalbrain
Thalamus
Caudatenucleus
ParietalLobe
MedullaOblongata
CingulateCortex
OccipitalLobe
TemporalLobe
SubthalamicNucleus
Pons
GlobusPallidus
Cerebellum
CerebellumPeduncles
CD34+
CD105+_Endothelial
721_B_lymphoblasts
CD19+_BCells(neg._sel.)
BDCA4+_DentriticCells
CD8+_Tcells
CD4+_Tcells
CD56+_NKCells
CD33+_Myeloid
CD14+_Monocytes
WholeBlood
Mean(7)
3xM
10xM

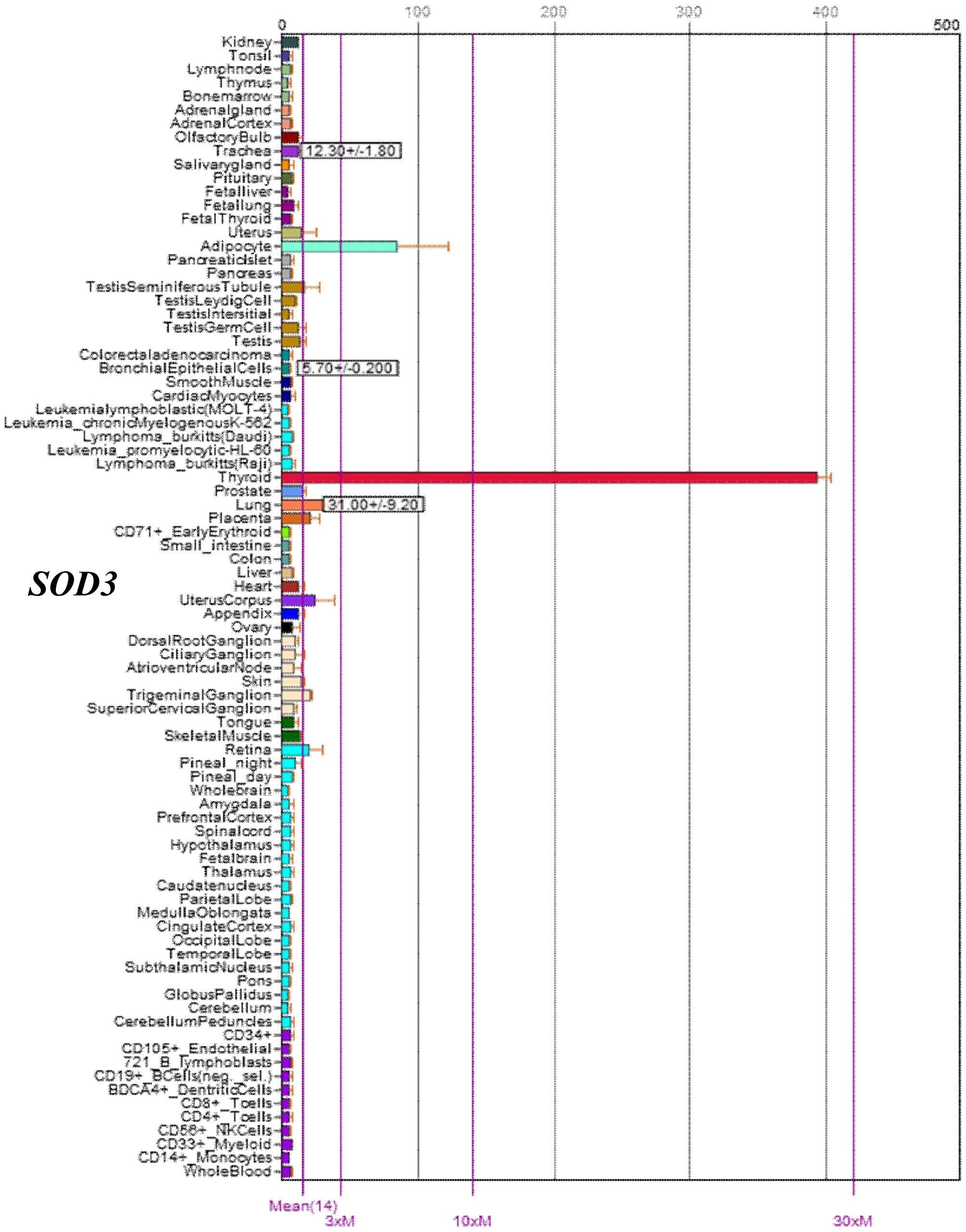
0
100
200
300
400
500
Kidney
Tonsil
Lymphnode
Thymus
Bonemarrow
Adrenalgland
AdrenalCortex
OlfactoryBulb
Trachea
12.30+/-1.80
Salivarygland
Pituitary
Fetalliver
Fetallung
FetalThyroid
Uterus
Adipocyte
Pancreaticlslet
Pancreas
TestisSeminiferousTubule
TestisLeydigCell
TestisIntersitial
TestisGermCell
Testis
Colorectaladenocarcinoma
BronchialEpithelialCells
5.70+/-0.200
SmoothMuscle
CardiacMyocytes
Leukemialymphoblastic(MOLT-4)
Leukemia_chronicMyelogenousK-562
Lymphoma_burkitts(Daudi)
Leukemia_promyelocytic-HL-60
Lymphoma_burkitts(Raji)
Thyroid
Prostate
Lung
31.00+/-9.20
Placenta
CD71+_EarlyErythroid
Small_intestine
Colon
Liver
Heart
UterusCorpus
Appendix
Ovary
DorsalRootGanglion
CiliaryGanglion
AtrioventricularNode
Skin
TrigeminalGanglion
SuperiorCervicalGanglion
Tongue
SkeletalMuscle
Retina
Pineal_night
Pineal_day
WholeBrain
Amygdala
PrefrontalCortex
Spinalcord
Hypothalamus
Fetalbrain
Thalamus
Caudatenucleus
ParietalLobe
MedullaOblongata
CingulateCortex
OccipitalLobe
TemporalLobe
SubthalamicNucleus
Pons
GlobusPallidus
Cerebellum
CerebellumPeduncles
CD34+
CD105+_Endothelial
721_B_lymphoblasts
CD19+_BCells(neg._sel.)
BDCA4+_DentriticCells
CD8+_Tcells
CD4+_Tcells
CD56+_NKCells
CD33+_Myeloid
CD14+_Monocytes
WholeBlood
Mean(14)
3xM
10xM
30xM

SOD3

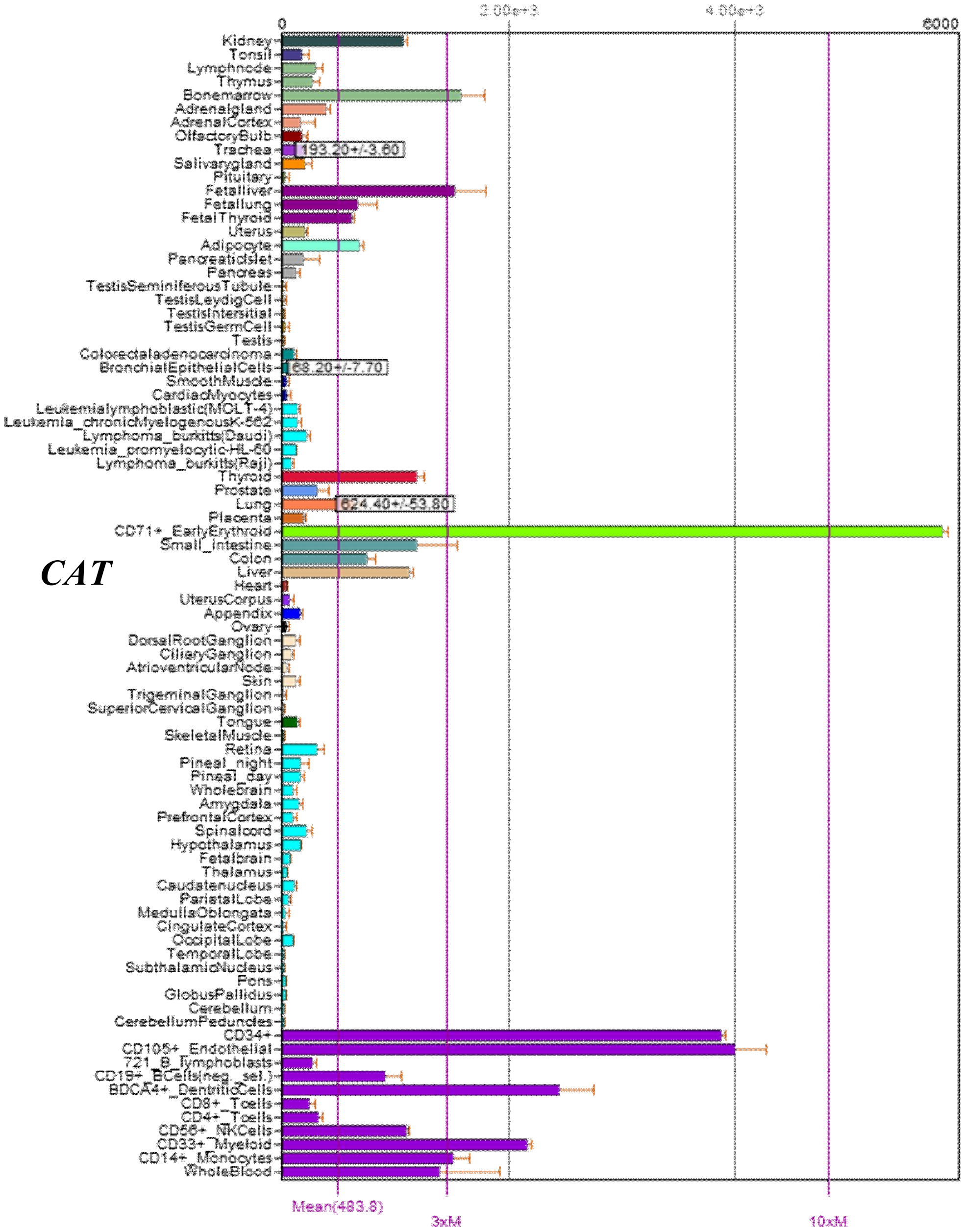
0
2.00e+3
4.00e+3
6000
Kidney
Tonsil
Lymphnode
Thymus
Bonemarrow
Adrenalgland
AdrenalCortex
OlfactoryBulb
Trachea
193.20+/-3.60
Salivarygland
Pituitary
Fetalliver
Fetallung
FetalThyroid
Uterus
Adipocyte
Pancreaticlslet
Pancreas
TestisSeminiferousTubule
TestisLeydigCell
TestisIntersitial
TestisGermCell
Testis
Colorectaladenocarcinoma
BronchialEpithelialCells
68.20+/-7.70
SmoothMuscle
CardiacMyocytes
Leukemialymphoblastic(MOLT-4)
Leukemia_chronicMyelogenousK-562
Lymphoma_burkitts(Daudi)
Leukemia_promyelocytic-HL-60
Lymphoma_burkitts(Raji)
Thyroid
Prostate
Lung
624.40+/-53.80
Placenta
CD71+_EarlyErythroid
Small_intestine
Colon
Liver
Heart
UterusCorpus
Appendix
Ovary
DorsalRootGanglion
CiliaryGanglion
AtrioventricularNode
Skin
TrigeminalGanglion
SuperiorCervicalGanglion
Tongue
SkeletalMuscle
Retina
Pineal_night
Pineal_day
WholeBrain
Amygdala
PrefrontalCortex
Spinalcord
Hypothalamus
Fetalbrain
Thalamus
Caudatenucleus
ParietalLobe
MedullaOblongata
CingulateCortex
OccipitalLobe
TemporalLobe
SubthalamicNucleus
Pons
GlobusPallidus
Cerebellum
CerebellumPeduncles
CD34+
CD105+_Endothelial
721_B_lymphoblasts
CD19+_BCells(neg._sel.)
BDCA4+_DentriticCells
CD8+_Tcells
CD4+_Tcells
CD56+_NKCells
CD33+_Myeloid
CD14+_Monocytes
WholeBlood
Mean(483.8)
3xM
10xM

CAT

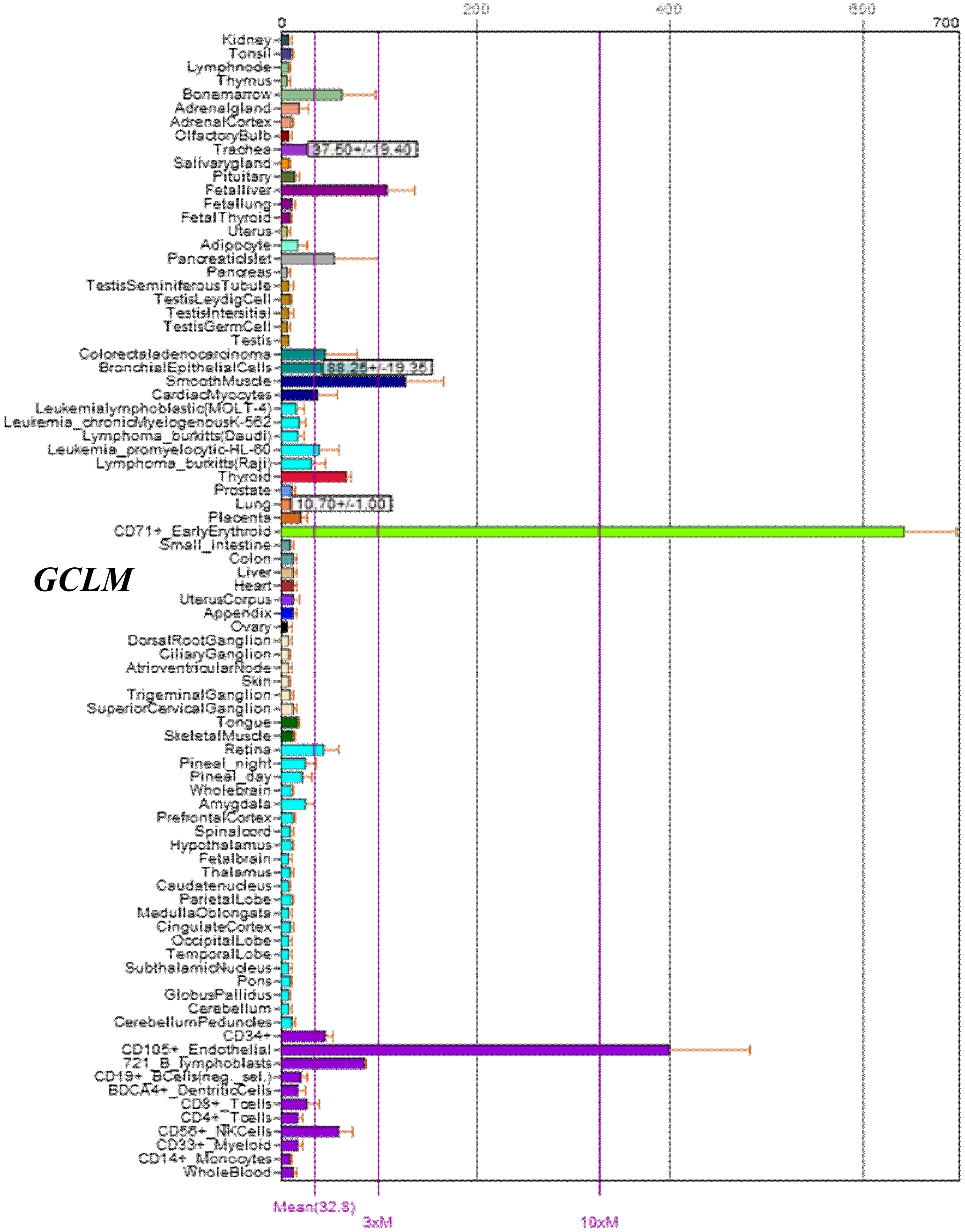

GCLM
0
200
400
600
700
Kidney
Tonsil
Lymphnode
Thymus
Bonemarrow
Adrenalgland
AdrenalCortex
OlfactoryBulb
Trachea
37.50+/-19.40
Salivarygland
Pituitary
Fetalliver
Fetallung
FetalThyroid
Uterus
Adipocyte
Pancreaticlslet
Pancreas
TestisSeminiferousTubule
TestisLeydigCell
TestisIntersitial
TestisGermCell
Testis
Colorectaladenocarcinoma
BronchialEpithelialCells
88.25+/-19.35
SmoothMuscle
CardiacMyocytes
Leukemialymphoblastic(MOLT-4)
Leukemia_chronicMyelogenousK-562
Lymphoma_burkitts(Daudi)
Leukemia_promyelocytic-HL-60
Lymphoma_burkitts(Raji)
Thyroid
Prostate
Lung
10.70+/-1.00
Placenta
CD71+_EarlyErythroid
Small_intestine
Colon
Liver
Heart
UterusCorpus
Appendix
Ovary
DorsalRootGanglion
CiliaryGanglion
AtrioventricularNode
Skin
TrigeminalGanglion
SuperiorCervicalGanglion
Tongue
SkeletalMuscle
Retina
Pineal_night
Pineal_day
WholeBrain
Amygdala
PrefrontalCortex
Spinalcord
Hypothalamus
Fetalbrain
Thalamus
Caudatenucleus
ParietalLobe
MedullaOblongata
CingulateCortex
OccipitalLobe
TemporalLobe
SubthalamicNucleus
Pons
GlobusPallidus
Cerebellum
CerebellumPeduncles
CD34+
CD105+_Endothelial
721_B_lymphoblasts
CD19+_BCells(neg._sel.)
BDCA4+_DentriticCells
CD8+_Tcells
CD4+_Tcells
CD56+_NKCells
CD33+_Myeloid
CD14+_Monocytes
WholeBlood
Mean(32.8)
3xM
10xM

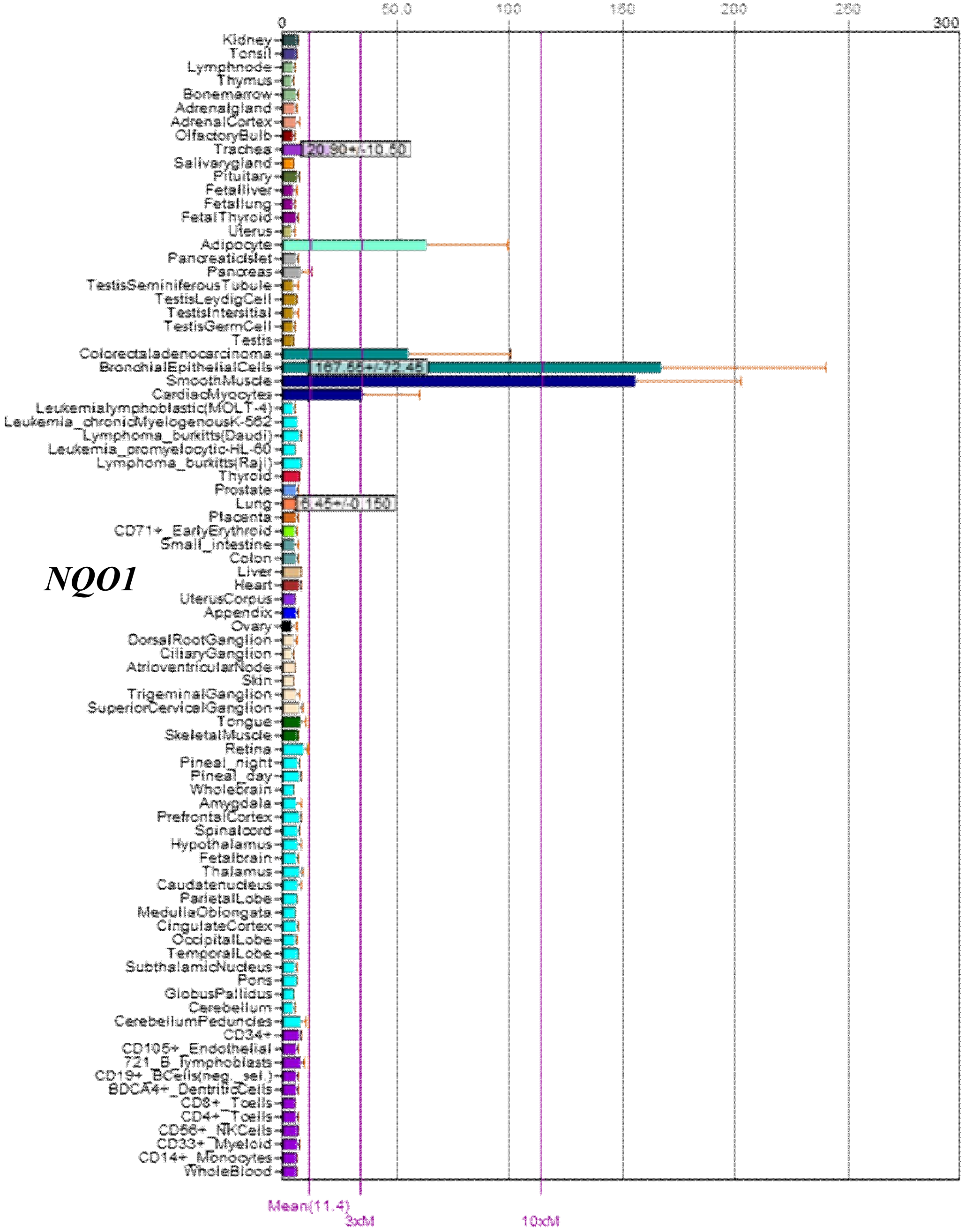

NQO1
0
50.0
100
150
200
250
300
Kidney
Tonsil
Lymphnode
Thymus
Bonemarrow
Adrenalgland
AdrenalCortex
OlfactoryBulb
Trachea
20.90+/-10.50
Salivarygland
Pituitary
Fetalliver
Fetallung
FetalThyroid
Uterus
Adipocyte
Pancreaticlslet
Pancreas
TestisSeminiferousTubule
TestisLeydigCell
TestisIntersitial
TestisGermCell
Testis
Colorectaladenocarcinoma
BronchialEpithelialCells
167.55+/-72.45
SmoothMuscle
CardiacMyocytes
Leukemialymphoblastic(MOLT-4)
Leukemia_chronicMyelogenousK-562
Lymphoma_burkitts(Daudi)
Leukemia_promyelocytic-HL-60
Lymphoma_burkitts(Raji)
Thyroid
Prostate
Lung
6.45+/-0.150
Placenta
CD71+_EarlyErythroid
Small_intestine
Colon
Liver
Heart
UterusCorpus
Appendix
Ovary
DorsalRootGanglion
CiliaryGanglion
AtrioventricularNode
Skin
TrigeminalGanglion
SuperiorCervicalGanglion
Tongue
SkeletalMuscle
Retina
Pineal_night
Pineal_day
WholeBrain
Amygdala
PrefrontalCortex
Spinalcord
Hypothalamus
Fetalbrain
Thalamus
Caudatenucleus
ParietalLobe
MedullaOblongata
CingulateCortex
OccipitalLobe
TemporalLobe
SubthalamicNucleus
Pons
GlobusPallidus
Cerebellum
CerebellumPeduncles
CD34+
CD105+_Endothelial
721_B_lymphoblasts
CD19+_BCells(neg._sel.)
BDCA4+_DentriticCells
CD8+_Tcells
CD4+_Tcells
CD56+_NKCells
CD33+_Myeloid
CD14+_Monocytes
WholeBlood
Mean(11.4)
3xM
10xM

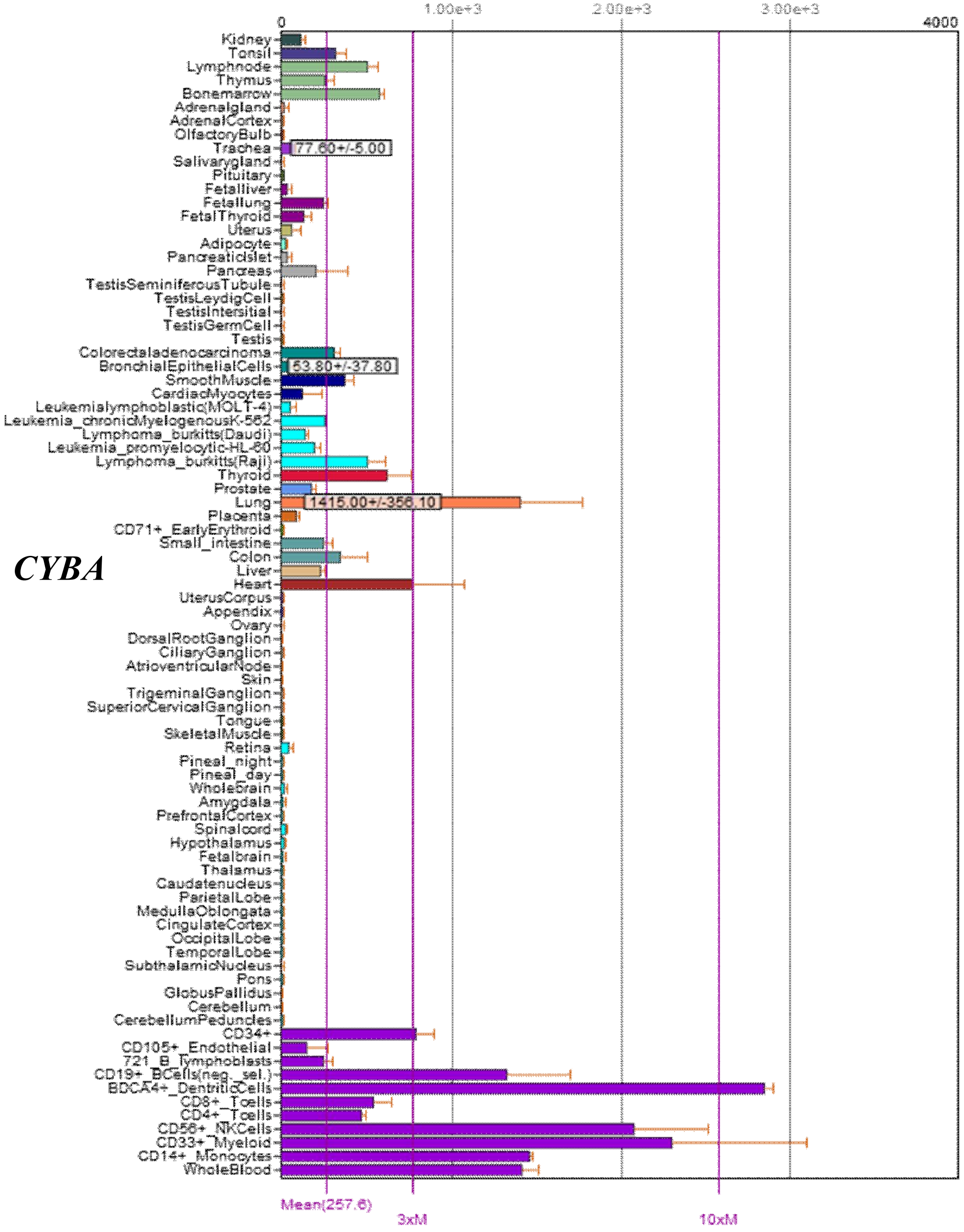
0
1.00e+3
2.00e+3
3.00e+3
4000
Kidney
Tonsil
Lymphnode
Thymus
Bonemarrow
Adrenalgland
AdrenalCortex
OlfactoryBulb
Trachea
77.60+/-5.00
Salivarygland
Pituitary
Fetalliver
Fetallung
FetalThyroid
Uterus
Adipocyte
Pancreaticlslet
Pancreas
TestisSeminiferousTubule
TestisLeydigCell
TestisIntersitial
TestisGermCell
Testis
Colorectaladenocarcinoma
BronchialEpithelialCells
53.80+/-37.80
SmoothMuscle
CardiacMyocytes
Leukemialymphoblastic(MOLT-4)
Leukemia_chronicMyelogenousK-562
Lymphoma_burkitts(Daudi)
Leukemia_promyelocytic-HL-60
Lymphoma_burkitts(Raji)
Thyroid
Prostate
Lung
1415.00+/-356.10
Placenta
CD71+_EarlyErythroid
Small_intestine
Colon
Liver
Heart
UterusCorpus
Appendix
Ovary
DorsalRootGanglion
CiliaryGanglion
AtrioventricularNode
Skin
TrigeminalGanglion
SuperiorCervicalGanglion
Tongue
SkeletalMuscle
Retina
Pineal_night
Pineal_day
Wholebrain
Amygdala
PrefrontalCortex
Spinalcord
Hypothalamus
Fetalbrain
Thalamus
Caudatenucleus
ParietalLobe
MedullaOblongata
CingulateCortex
OccipitalLobe
TemporalLobe
SubthalamicNucleus
Pons
GlobusPallidus
Cerebellum
CerebellumPeduncles
CD34+
CD105+_Endothelial
721_B_lymphoblasts
CD19+_BCells(neg._sel.)
BDCA4+_DentriticCells
CD8+_Tcells
CD4+_Tcells
CD56+_NKCells
CD33+_Myeloid
CD14+_Monocytes
WholeBlood
Mean(257.6)
3xM
10xM

CYBA

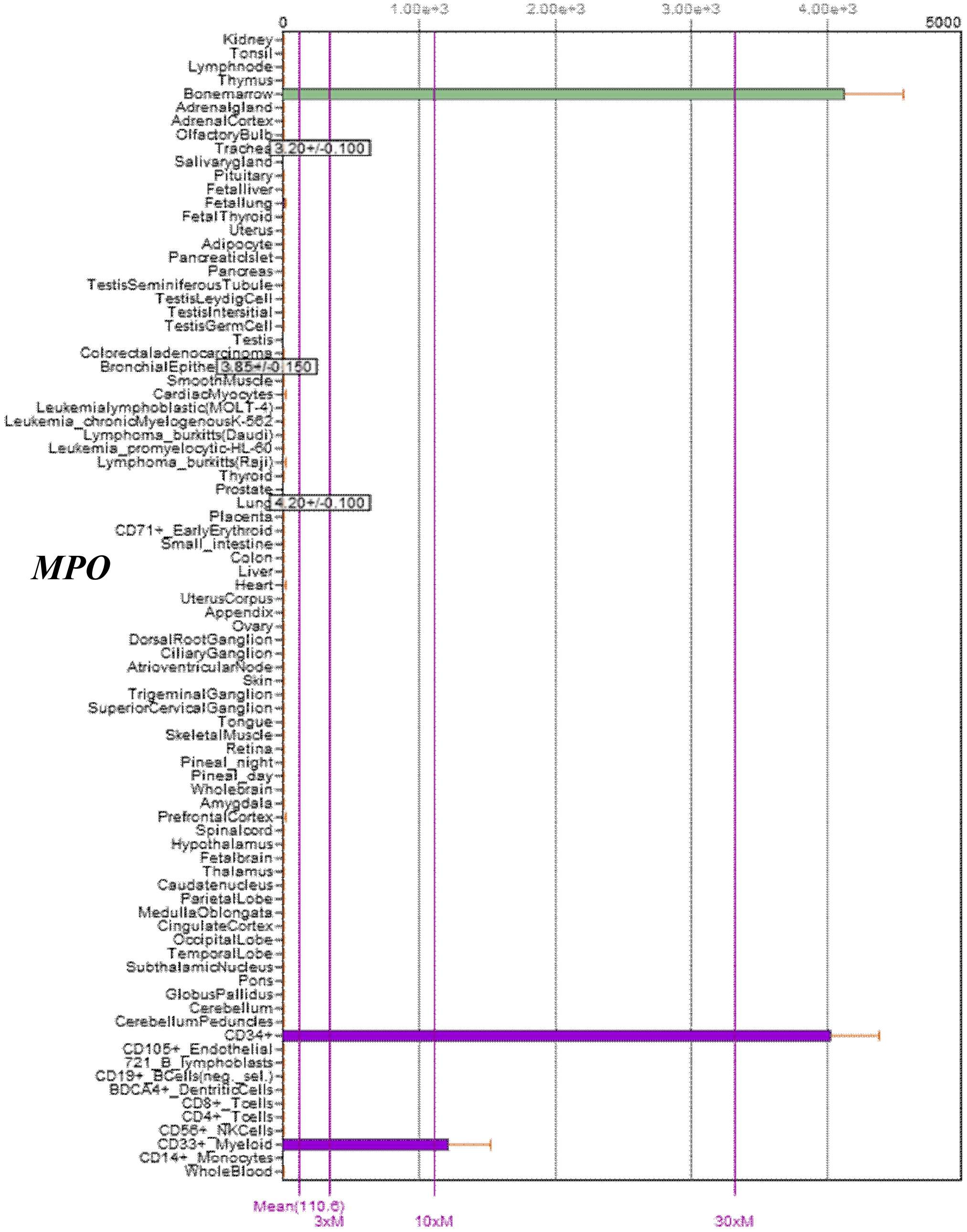
0
1.00e+3
2.00e+3
3.00e+3
4.00e+3
5000
Kidney
Tonsil
Lymphnode
Thymus
Bonemarrow
Adrenalgland
AdrenalCortex
OlfactoryBulb
Trachea
3.20+/-0.100
Salivarygland
Pituitary
Fetalliver
Fetallung
FetalThyroid
Uterus
Adipocyte
Pancreaticlslet
Pancreas
TestisSeminiferousTubule
TestisLeydigCell
TestisIntersitial
TestisGermCell
Testis
Colorectaladenocarcinoma
BronchialEpithe
3.85+/-0.150
SmoothMuscle
CardiacMyocytes
Leukemialymphoblastic(MOLT-4)
Leukemia_chronicMyelogenousK-562
Lymphoma_burkitts(Daudi)
Leukemia_promyelocytic-HL-60
Lymphoma_burkitts(Raji)
Thyroid
Prostate
Lung
4.20+/-0.100
Placenta
CD71+_EarlyErythroid
Small_intestine
Colon
Liver
Heart
UterusCorpus
Appendix
Ovary
DorsalRootGanglion
CiliaryGanglion
AtrioventricularNode
Skin
TrigeminalGanglion
SuperiorCervicalGanglion
Tongue
SkeletalMuscle
Retina
Pineal_night
Pineal_day
Wholebrain
Amygdala
PrefrontalCortex
Spinalcord
Hypothalamus
Fetalbrain
Thalamus
Caudatenucleus
ParietalLobe
MedullaOblongata
CingulateCortex
OccipitalLobe
TemporalLobe
SubthalamicNucleus
Pons
GlobusPallidus
Cerebellum
CerebellumPeduncles
CD34+
CD105+_Endothelial
721_B_lymphoblasts
CD19+_BCells(neg._sel.)
BDCA4+_DentriticCells
CD8+_Tcells
CD4+_Tcells
CD56+_NKCells
CD33+_Myeloid
CD14+_Monocytes
WholeBlood
Mean(110.6)
3xM
10xM
30xM

MPO

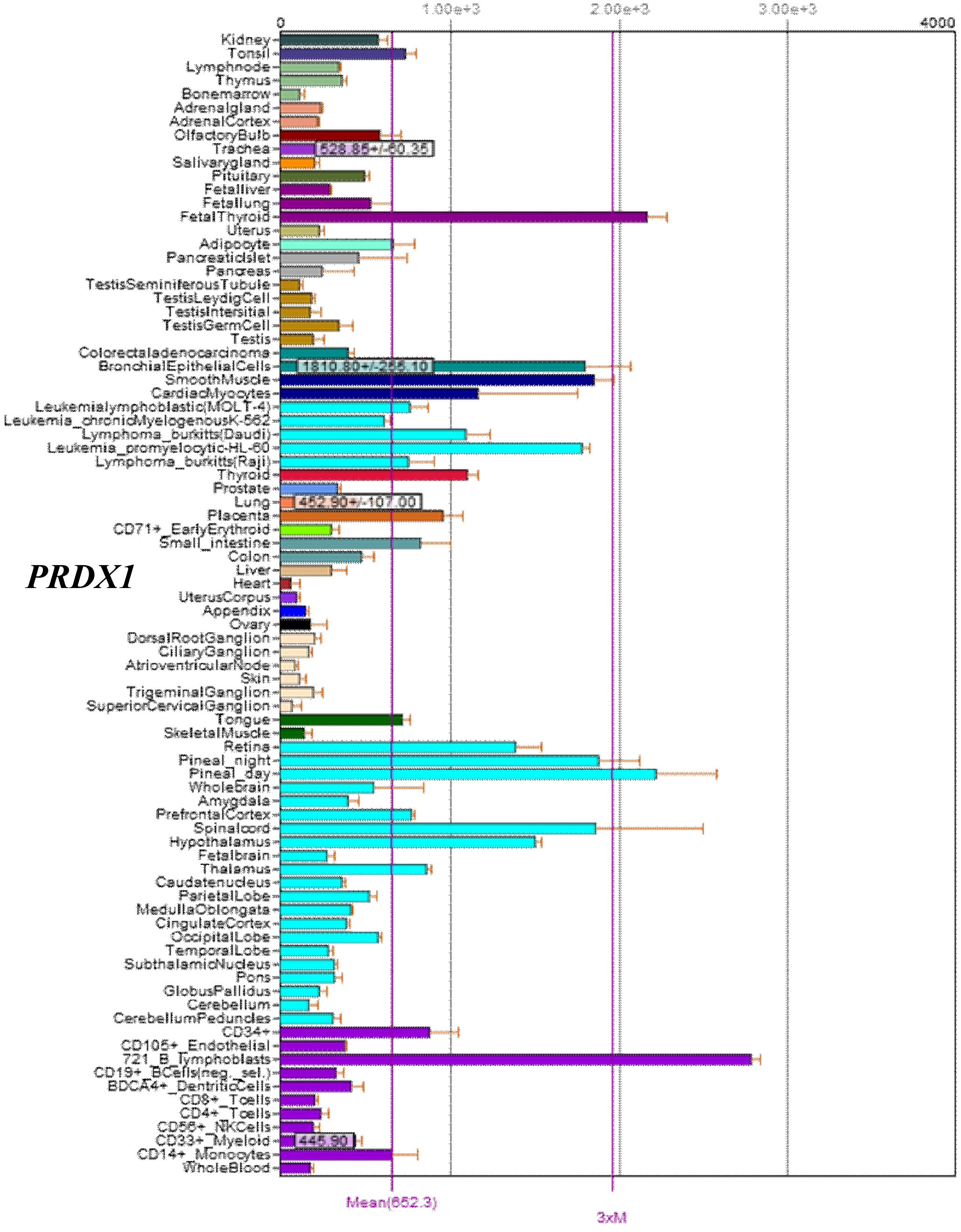
PRDX1
0
1.00e+3
2.00e+3
3.00e+3
4000
Kidney
Tonsil
Lymphnode
Thymus
Bonemarrow
Adrenalgland
AdrenalCortex
OlfactoryBulb
Trachea
528.85+/-60.35
Salivarygland
Pituitary
Fetalliver
Fetallung
FetalThyroid
Uterus
Adipocyte
Pancreaticlslet
Pancreas
TestisSeminiferousTubule
TestisLeydigCell
TestisIntersitial
TestisGermCell
Testis
Colorectaladenocarcinoma
BronchialEpithelialCells
1810.80+/-255.10
SmoothMuscle
CardiacMyocytes
Leukemialymphoblastic(MOLT-4)
Leukemia_chronicMyelogenousK-562
Lymphoma_burkitts(Daudi)
Leukemia_promyelocytic-HL-60
Lymphoma_burkitts(Raji)
Thyroid
Prostate
Lung
452.90+/-107.00
Placenta
CD71+_EarlyErythroid
Small_intestine
Colon
Liver
Heart
UterusCorpus
Appendix
Ovary
DorsalRootGanglion
CiliaryGanglion
AtrioventricularNode
Skin
TrigeminalGanglion
SuperiorCervicalGanglion
Tongue
SkeletalMuscle
Retina
Pineal_night
Pineal_day
WholeBrain
Amygdala
PrefrontalCortex
Spinalcord
Hypothalamus
Fetalbrain
Thalamus
Caudatenucleus
ParietalLobe
MedullaOblongata
CingulateCortex
OccipitalLobe
TemporalLobe
SubthalamicNucleus
Pons
GlobusPallidus
Cerebellum
CerebellumPeduncles
CD34+
CD105+_Endothelial
721_B_lymphoblasts
CD19+_BCells(neg._sel.)
BDCA4+_DentriticCells
CD8+_Tcells
CD4+_Tcells
CD56+_NKCells
CD33+_Myeloid
445.90
CD14+_Monocytes
WholeBlood
Mean(652.3)
3xM

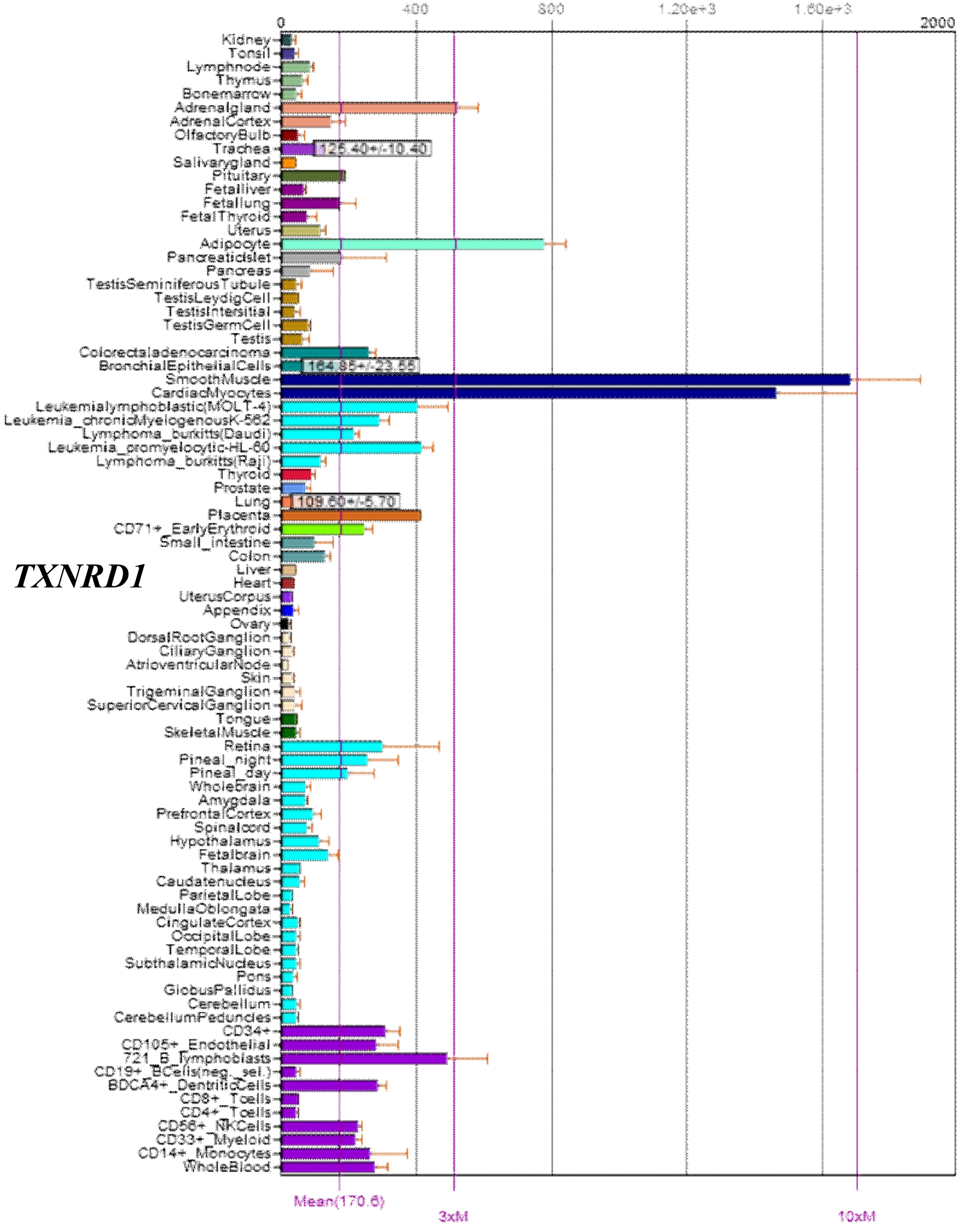
TXNRD1
0
400
800
1.20e+3
1.60e+3
2000
Kidney
Tonsil
Lymphnode
Thymus
Bonemarrow
Adrenalgland
AdrenalCortex
OlfactoryBulb
Trachea
125.40+/-10.40
Salivarygland
Pituitary
Fetalliver
Fetallung
FetalThyroid
Uterus
Adipocyte
Pancreaticlslet
Pancreas
TestisSeminiferousTubule
TestisLeydigCell
TestisIntersitial
TestisGermCell
Testis
Colorectaladenocarcinoma
BronchialEpithelialCells
164.85+/-23.55
SmoothMuscle
CardiacMyocytes
Leukemialymphoblastic(MOLT-4)
Leukemia_chronicMyelogenousK-562
Lymphoma_burkitts(Daudi)
Leukemia_promyelocytic-HL-60
Lymphoma_burkitts(Raji)
Thyroid
Prostate
Lung
109.60+/-5.70
Placenta
CD71+_EarlyErythroid
Small_intestine
Colon
Liver
Heart
UterusCorpus
Appendix
Ovary
DorsalRootGanglion
CiliaryGanglion
AtrioventricularNode
Skin
TrigeminalGanglion
SuperiorCervicalGanglion
Tongue
SkeletalMuscle
Retina
Pineal_night
Pineal_day
WholeBrain
Amygdala
PrefrontalCortex
Spinalcord
Hypothalamus
Fetalbrain
Thalamus
Caudatenucleus
ParietalLobe
MedullaOblongata
CingulateCortex
OccipitalLobe
TemporalLobe
SubthalamicNucleus
Pons
GlobusPallidus
Cerebellum
CerebellumPeduncles
CD34+
CD105+_Endothelial
721_B_lymphoblasts
CD19+_BCells(neg._sel.)
BDCA4+_DentriticCells
CD8+_Tcells
CD4+_Tcells
CD56+_NKCells
CD33+_Myeloid
CD14+_Monocytes
WholeBlood
Mean(170.6)
3xM
10xM

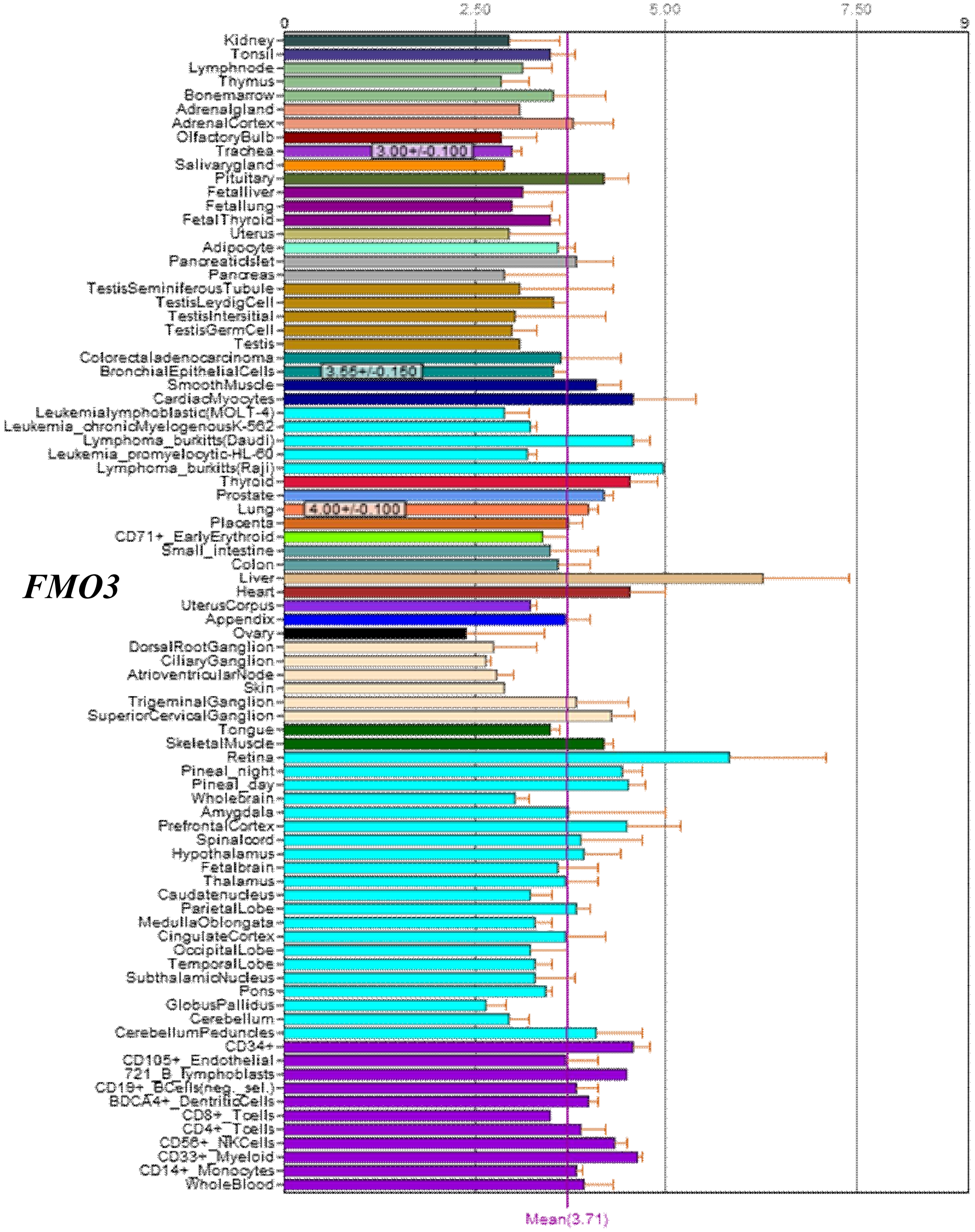
FMO3
0
2.50
5.00
7.50
9
Kidney
Tonsil
Lymphnode
Thymus
Bonemarrow
Adrenalgland
AdrenalCortex
OlfactoryBulb
Trachea
Salivarygland
Pituitary
Fetalliver
Fetallung
FetalThyroid
Uterus
Adipocyte
Pancreaticlslet
Pancreas
TestisSeminiferousTubule
TestisLeydigCell
TestisIntersitial
TestisGermCell
Testis
Colorectaladenocarcinoma
BronchialEpithelialCells
SmoothMuscle
CardiacMyocytes
Leukemialymphoblastic(MOLT-4)
Leukemia_chronicMyelogenousK-562
Lymphoma_burkitts(Daudi)
Leukemia_promyelocytic-HL-60
Lymphoma_burkitts(Raji)
Thyroid
Prostate
Lung
Placenta
CD71+_EarlyErythroid
Small_intestine
Colon
Liver
Heart
UterusCorpus
Appendix
Ovary
DorsalRootGanglion
CiliaryGanglion
AtrioventricularNode
Skin
TrigeminalGanglion
SuperiorCervicalGanglion
Tongue
SkeletalMuscle
Retina
Pineal_night
Pineal_day
WholeBrain
Amygdala
PrefrontalCortex
Spinalcord
Hypothalamus
Fetalbrain
Thalamus
Caudatenucleus
ParietalLobe
MedullaOblongata
CingulateCortex
OccipitalLobe
TemporalLobe
SubthalamicNucleus
Pons
GlobusPallidus
Cerebellum
CerebellumPeduncles
CD34+
CD105+_Endothelial
721_B_lymphoblasts
CD19+_BCells(neg._sel.)
BDCA4+_DentriticCells
CD8+_Tcells
CD4+_Tcells
CD56+_NKCells
CD33+_Myeloid
CD14+_Monocytes
WholeBlood
3.00+/-0.100
3.55+/-0.150
4.00+/-0.100
Mean(3.71)

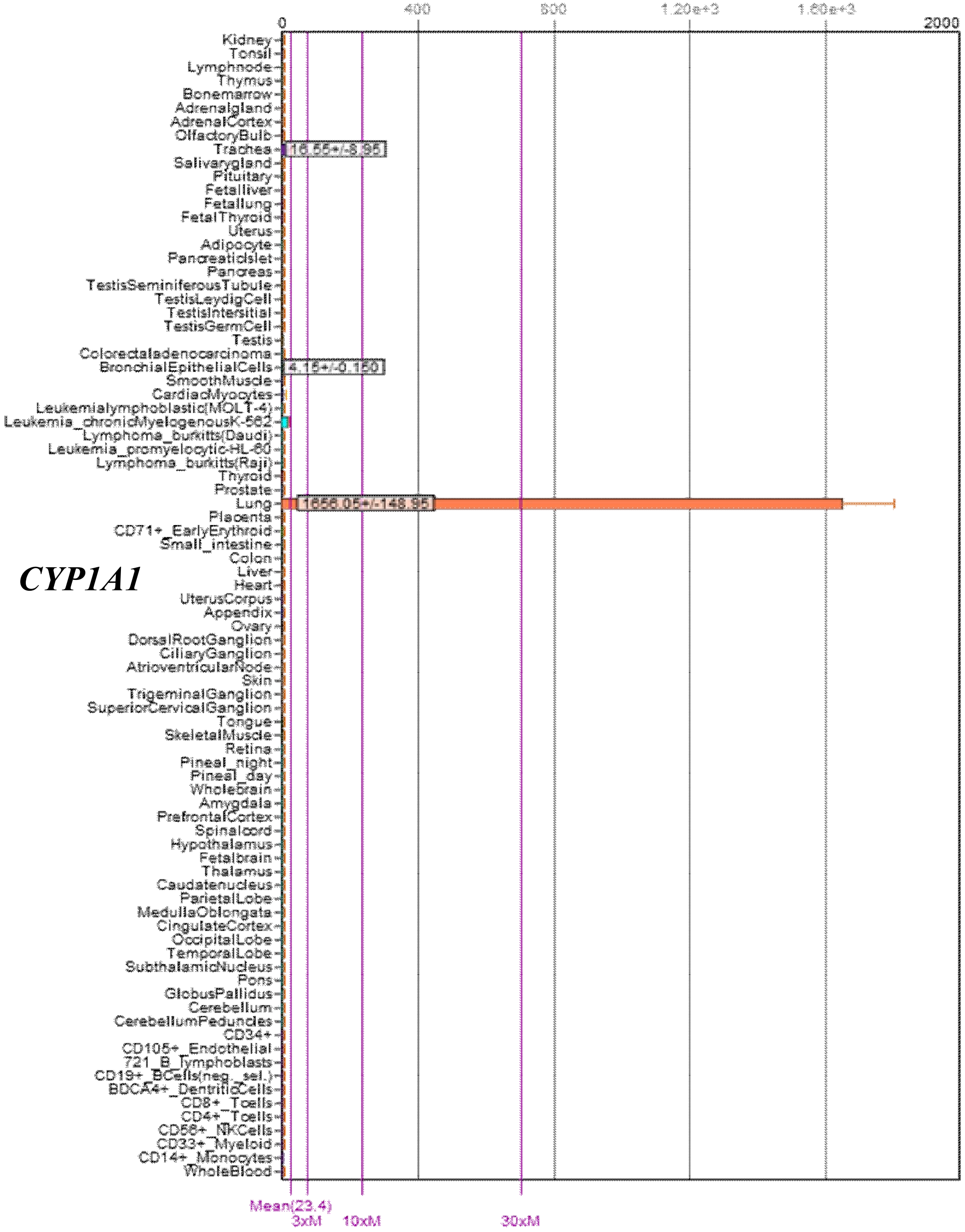
CYP1A1
0
400
800
1.20e+3
1.60e+3
2000
Kidney
Tonsil
Lymphnode
Thymus
Bonemarrow
Adrenalgland
AdrenalCortex
OlfactoryBulb
Trachea
Salivarygland
Pituitary
Fetalliver
Fetallung
FetalThyroid
Uterus
Adipocyte
Pancreaticlslet
Pancreas
TestisSeminiferousTubule
TestisLeydigCell
TestisIntersitial
TestisGermCell
Testis
Colorectaladenocarcinoma
BronchialEpithelialCells
SmoothMuscle
CardiacMyocytes
Leukemialymphoblastic(MOLT-4)
Leukemia_chronicMyelogenousK-562
Lymphoma_burkitts(Daudi)
Leukemia_promyelocytic-HL-60
Lymphoma_burkitts(Raji)
Thyroid
Prostate
Lung
Placenta
CD71+_EarlyErythroid
Small_intestine
Colon
Liver
Heart
UterusCorpus
Appendix
Ovary
DorsalRootGanglion
CiliaryGanglion
AtrioventricularNode
Skin
TrigeminalGanglion
SuperiorCervicalGanglion
Tongue
SkeletalMuscle
Retina
Pineal_night
Pineal_day
WholeBrain
Amygdala
PrefrontalCortex
Spinalcord
Hypothalamus
Fetalbrain
Thalamus
Caudatenucleus
ParietalLobe
MedullaOblongata
CingulateCortex
OccipitalLobe
TemporalLobe
SubthalamicNucleus
Pons
GlobusPallidus
Cerebellum
CerebellumPeduncles
CD34+
CD105+_Endothelial
721_B_lymphoblasts
CD19+_BCells(neg._sel.)
BDCA4+_DentriticCells
CD8+_Tcells
CD4+_Tcells
CD56+_NKCells
CD33+_Myeloid
CD14+_Monocytes
WholeBlood
16.55+/-8.95
4.15+/-0.150
1656.05+/-148.95
Mean(23.4)
3xM
10xM
30xM

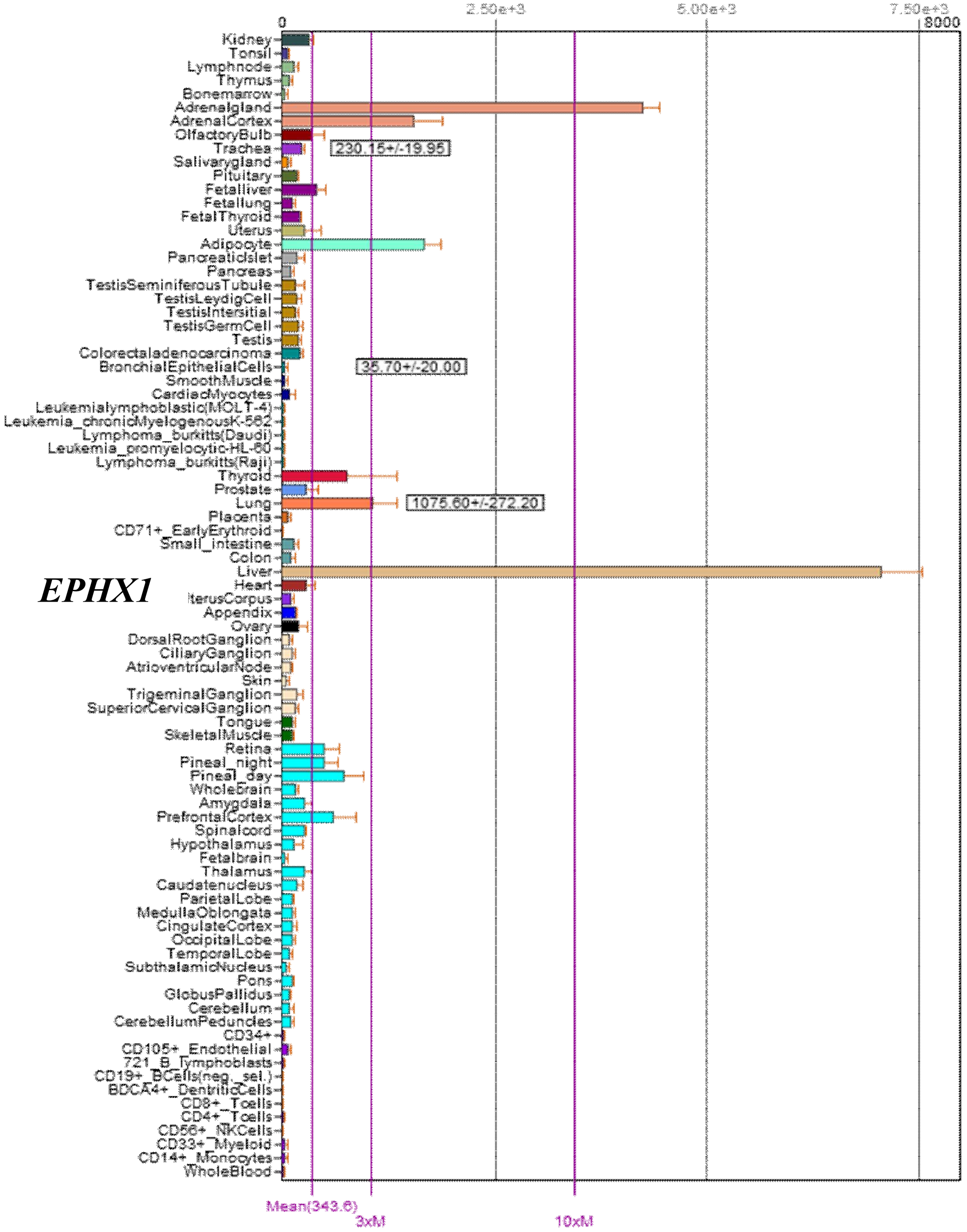

EPHX1
0
2.50e+3
5.00e+3
7.50e+3
8000
Kidney
Tonsil
Lymphnode
Thymus
Bonemarrow
Adrenalgland
AdrenalCortex
OlfactoryBulb
Trachea
Salivarygland
Pituitary
Fetalliver
Fetallung
FetalThyroid
Uterus
Adipocyte
PancreaticIslet
Pancreas
TestisSeminiferousTubule
TestisLeydigCell
TestisIntersitial
TestisGermCell
Testis
Colorectaladenocarcinoma
BronchialEpithelialCells
SmoothMuscle
CardiacMyocytes
Leukemialymphoblastic(MOLT-4)
Leukemia_chronicMyelogenousK-562
Lymphoma_burkitts(Daudi)
Leukemia_promyelocytic-HL-60
Lymphoma_burkitts(Raji)
Thyroid
Prostate
Lung
Placenta
CD71+_EarlyErythroid
Small_intestine
Colon
Liver
Heart
IterusCorpus
Appendix
Ovary
DorsalRootGanglion
CiliaryGanglion
AtrioventricularNode
Skin
TrigeminalGanglion
SuperiorCervicalGanglion
Tongue
SkeletalMuscle
Retina
Pineal_night
Pineal_day
Wholebrain
Amygdala
PrefrontalCortex
Spinalcord
Hypothalamus
Fetalbrain
Thalamus
Caudatenucleus
ParietalLobe
MedullaOblongata
CingulateCortex
OccipitalLobe
TemporalLobe
SubthalamicNucleus
Pons
GlobusPallidus
Cerebellum
CerebellumPeduncles
CD34+
CD105+_Endothelial
721_B_lymphoblasts
CD19+_BCells(neg._sel.)
BDCA4+_DentriticCells
CD8+_Tcells
CD4+_Tcells
CD56+_NKCells
CD33+_Myeloid
CD14+_Monocytes
WholeBlood
230.15+/-19.95
35.70+/-20.00
1075.60+/-272.20
Mean(343.6)
3xM
10xM

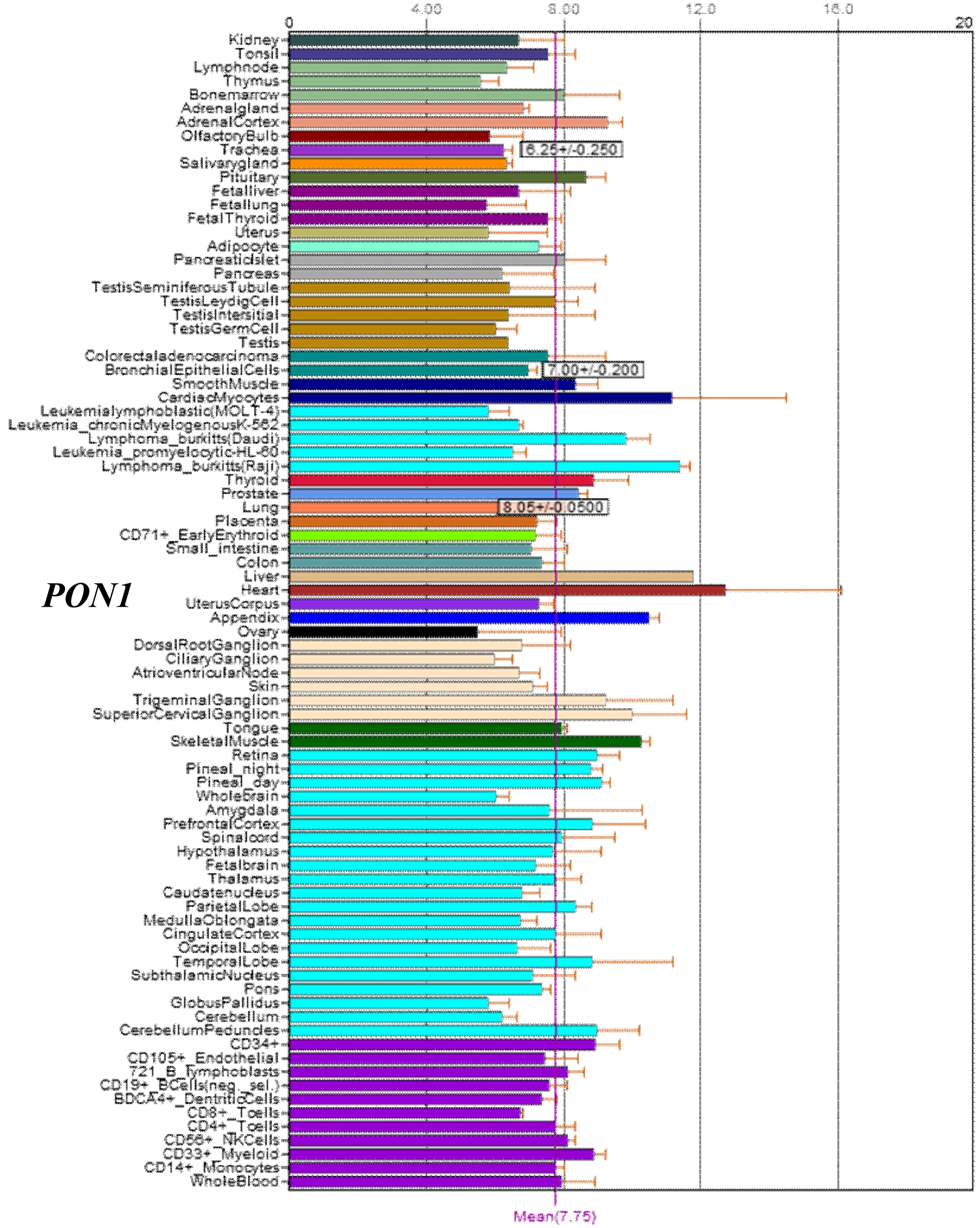

PON1
0
4.00
8.00
12.0
16.0
20
Kidney
Tonsil
Lymphnode
Thymus
Bonemarrow
Adrenalgland
AdrenalCortex
OlfactoryBulb
Trachea
Salivarygland
Pituitary
Fetalliver
Fetallung
FetalThyroid
Uterus
Adipocyte
PancreaticIslet
Pancreas
TestisSeminiferousTubule
TestisLeydigCell
TestisIntersitial
TestisGermCell
Testis
Colorectaladenocarcinoma
BronchialEpithelialCells
SmoothMuscle
CardiacMyocytes
Leukemialymphoblastic(MOLT-4)
Leukemia_chronicMyelogenousK-562
Lymphoma_burkitts(Daudi)
Leukemia_promyelocytic-HL-60
Lymphoma_burkitts(Raji)
Thyroid
Prostate
Lung
Placenta
CD71+_EarlyErythroid
Small_intestine
Colon
Liver
Heart
UterusCorpus
Appendix
Ovary
DorsalRootGanglion
CiliaryGanglion
AtrioventricularNode
Skin
TrigeminalGanglion
SuperiorCervicalGanglion
Tongue
SkeletalMuscle
Retina
Pineal_night
Pineal_day
Wholebrain
Amygdala
PrefrontalCortex
Spinalcord
Hypothalamus
Fetalbrain
Thalamus
Caudatenucleus
ParietalLobe
MedullaOblongata
CingulateCortex
OccipitalLobe
TemporalLobe
SubthalamicNucleus
Pons
GlobusPallidus
Cerebellum
CerebellumPeduncles
CD34+
CD105+_Endothelial
721_B_lymphoblasts
CD19+_BCells(neg._sel.)
BDCA4+_DentriticCells
CD8+_Tcells
CD4+_Tcells
CD56+_NKCells
CD33+_Myeloid
CD14+_Monocytes
WholeBlood
6.25+/-0.250
7.00+/-0.200
8.05+/-0.0500
Mean(7.75)

PON2

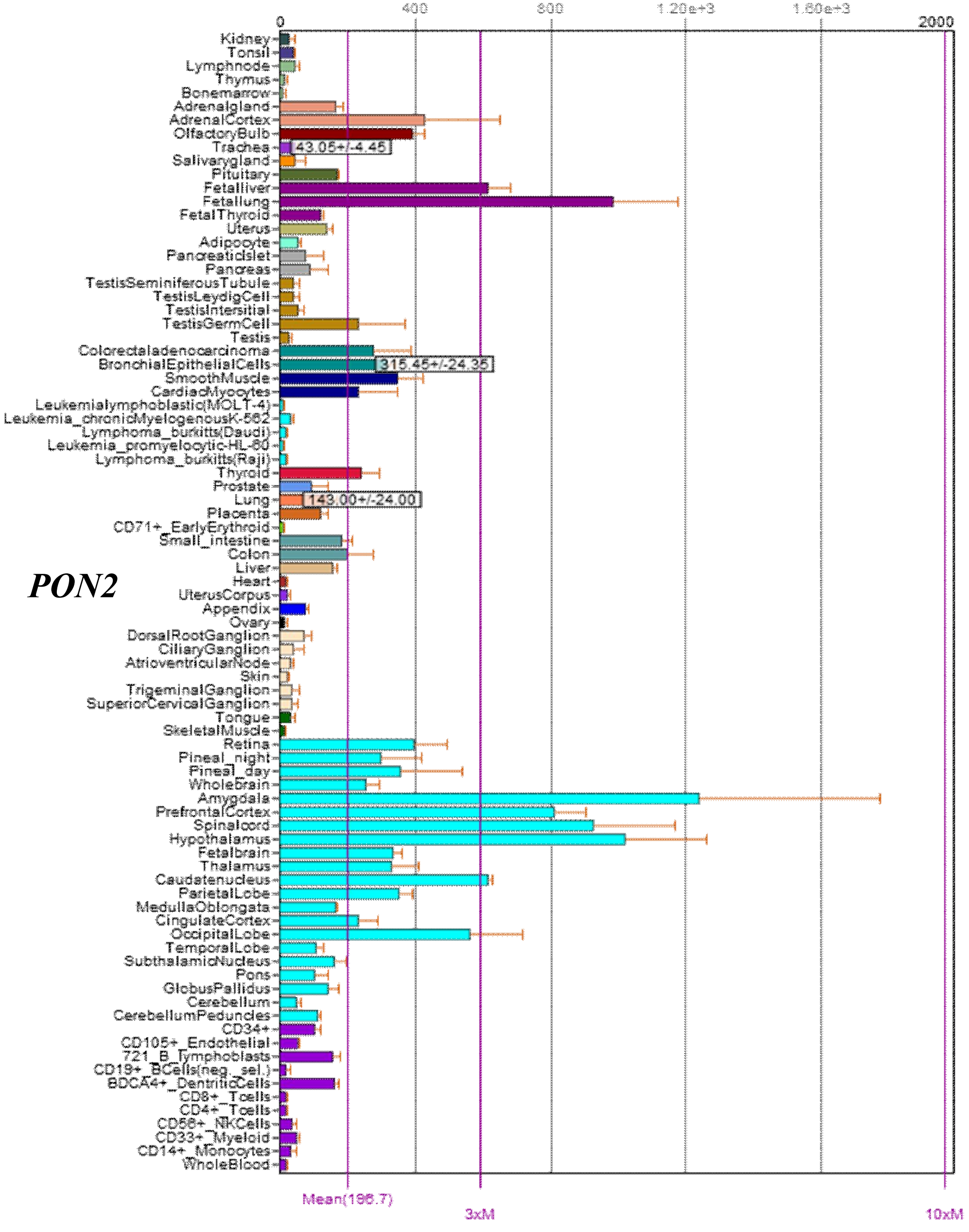

0
400
800
1.20e+3
1.60e+3
2000
Kidney
Tonsil
Lymphnode
Thymus
Bonemarrow
Adrenalgland
AdrenalCortex
OlfactoryBulb
Trachea
43.05+/-4.45
Salivarygland
Pituitary
Fetalliver
Fetallung
FetalThyroid
Uterus
Adipocyte
Pancreaticlslet
Pancreas
TestisSeminiferousTubule
TestisLeydigCell
TestisIntersitial
TestisGermCell
Testis
Colorectaladenocarcinoma
BronchialEpithelialCells
315.45+/-24.35
SmoothMuscle
CardiacMyocytes
Leukemialymphoblastic(MOLT-4)
Leukemia_chronicMyelogenousK-562
Lymphoma_burkitts(Daudi)
Leukemia_promyelocytic-HL-60
Lymphoma_burkitts(Raji)
Thyroid
Prostate
Lung
143.00+/-24.00
Placenta
CD71+_EarlyErythroid
Small_intestine
Colon
Liver
Heart
UterusCorpus
Appendix
Ovary
DorsalRootGanglion
CiliaryGanglion
AtrioventricularNode
Skin
TrigeminalGanglion
SuperiorCervicalGanglion
Tongue
SkeletalMuscle
Retina
Pineal_night
Pineal_day
Wholebrain
Amygdala
PrefrontalCortex
Spinalcord
Hypothalamus
Fetalbrain
Thalamus
Caudatenucleus
ParietalLobe
MedullaOblongata
CingulateCortex
OccipitalLobe
TemporalLobe
SubthalamicNucleus
Pons
GlobusPallidus
Cerebellum
CerebellumPeduncles
CD34+
CD105+_Endothelial
721_B_lymphoblasts
CD19+_BCells(neg._sel.)
BDCA4+_DentriticCells
CD8+_Tcells
CD4+_Tcells
CD56+_NKCells
CD33+_Myeloid
CD14+_Monocytes
WholeBlood
Mean(196.7)
3xM
10xM

GSTM1

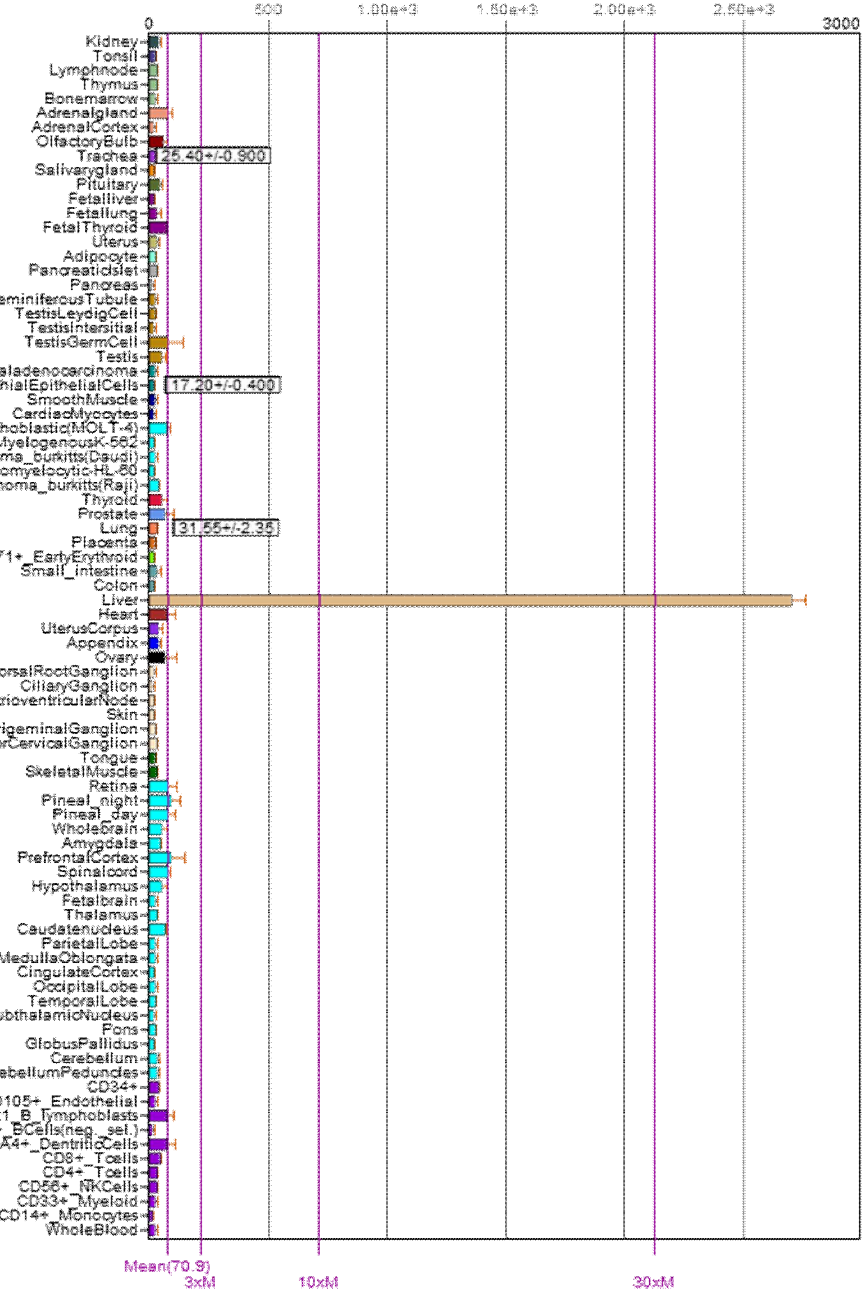

0
500
1.00e+3
1.50e+3
2.00e+3
2.50e+3
3000
Kidney
Tonsil
Lymphnode
Thymus
Bonemarrow
Adrenalgland
AdrenalCortex
OlfactoryBulb
Trachea
25.40+/-0.900
Salivarygland
Pituitary
Fetalliver
Fetallung
FetalThyroid
Uterus
Adipocyte
Pancreaticlslet
Pancreas
TestisSeminiferousTubule
TestisLeydigCell
TestisIntersitial
TestisGermCell
Testis
Colorectaladenocarcinoma
BronchialEpithelialCells
17.20+/-0.400
SmoothMuscle
CardiacMyocytes
Leukemialymphoblastic(MOLT-4)
Leukemia_chronicMyelogenousK-562
Lymphoma_burkitts(Daudi)
Leukemia_promyelocytic-HL-60
Lymphoma_burkitts(Raji)
Thyroid
Prostate
Lung
31.55+/-2.35
Placenta
CD71+_EarlyErythroid
Small_intestine
Colon
Liver
Heart
UterusCorpus
Appendix
Ovary
DorsalRootGanglion
CiliaryGanglion
AtrioventricularNode
Skin
TrigeminalGanglion
SuperiorCervicalGanglion
Tongue
SkeletalMuscle
Retina
Pineal_night
Pineal_day
Wholebrain
Amygdala
PrefrontalCortex
Spinalcord
Hypothalamus
Fetalbrain
Thalamus
Caudatenucleus
ParietalLobe
MedullaOblongata
CingulateCortex
OccipitalLobe
TemporalLobe
SubthalamicNucleus
Pons
GlobusPallidus
Cerebellum
CerebellumPeduncles
CD34+
CD105+_Endothelial
721_B_lymphoblasts
CD19+_BCells(neg._sel.)
BDCA4+_DentriticCells
CD8+_Tcells
CD4+_Tcells
CD56+_NKCells
CD33+_Myeloid
CD14+_Monocytes
WholeBlood
Mean(70.9)
3xM
10xM
30xM

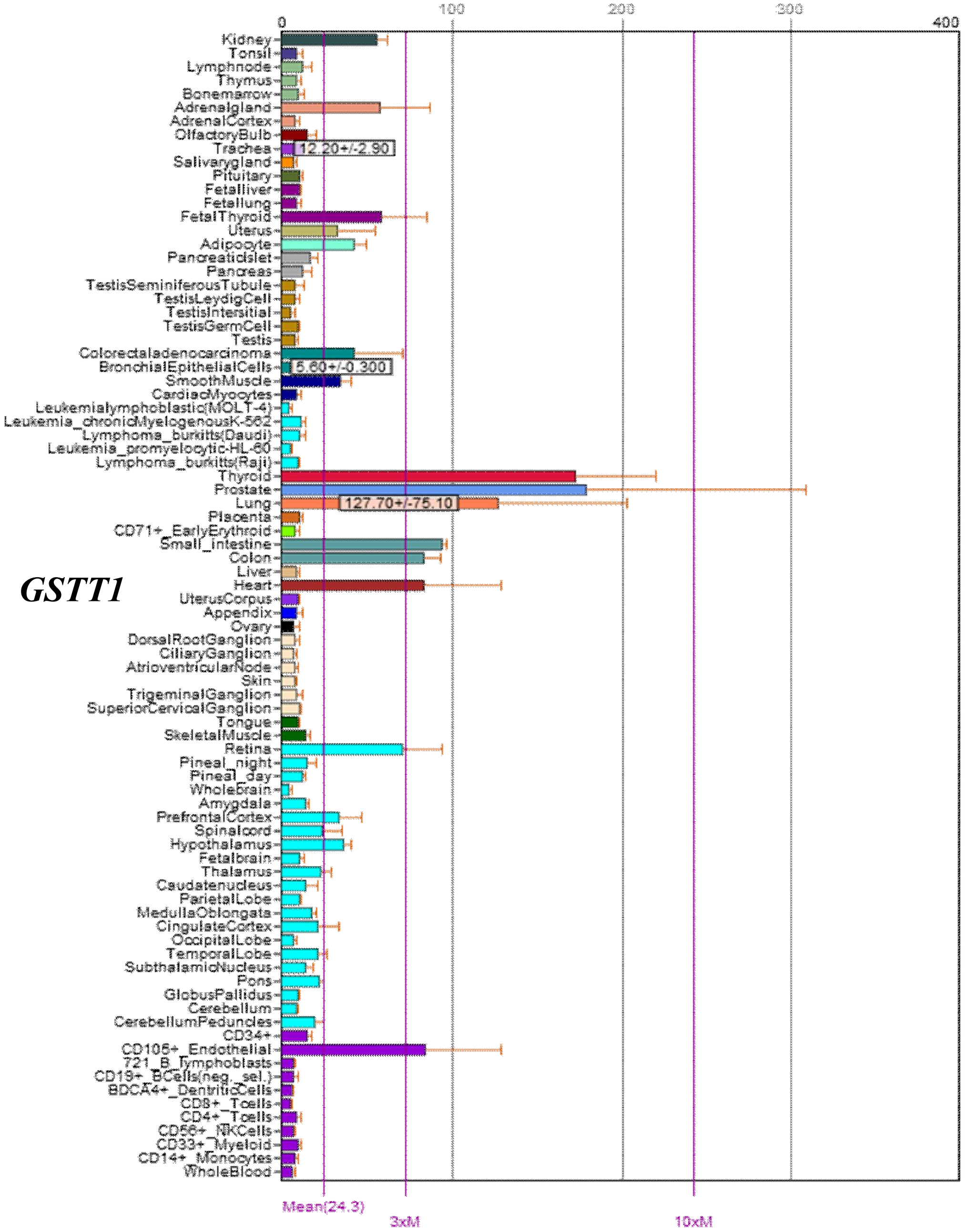

0
100
200
300
400
Kidney
Tonsil
Lymphnode
Thymus
Bonemarrow
Adrenalgland
AdrenalCortex
OlfactoryBulb
Trachea
12.20+/-2.90
Salivarygland
Pituitary
Fetalliver
Fetallung
FetalThyroid
Uterus
Adipocyte
Pancreaticislet
Pancreas
TestisSeminiferousTubule
TestisLeydigCell
TestisIntersitial
TestisGermCell
Testis
Colorectaladenocarcinoma
BronchialEpithelialCells
5.60+/-0.300
SmoothMuscle
CardiacMyocytes
Leukemialymphoblastic(MOLT-4)
Leukemia_chronicMyelogenousK-562
Lymphoma_burkitts(Daudi)
Leukemia_promyelocytic-HL-60
Lymphoma_burkitts(Raji)
Thyroid
Prostate
Lung
127.70+/-75.10
Placenta
CD71+_EarlyErythroid
Small_intestine
Colon
Liver
Heart
UterusCorpus
Appendix
Ovary
DorsalRootGanglion
CiliaryGanglion
AtrioventricularNode
Skin
TrigeminalGanglion
SuperiorCervicalGanglion
Tongue
SkeletalMuscle
Retina
Pineal_night
Pineal_day
Wholebrain
Amygdala
PrefrontalCortex
Spinalcord
Hypothalamus
Fetalbrain
Thalamus
Caudatenucleus
ParietalLobe
MedullaOblongata
CingulateCortex
OccipitalLobe
TemporalLobe
SubthalamicNucleus
Pons
GlobusPallidus
Cerebellum
CerebellumPeduncles
CD34+
CD105+_Endothelial
721_B_lymphoblasts
CD19+_BCells(neg._sel.)
BDCA4+_DentriticCells
CD8+_Tcells
CD4+_Tcells
CD56+_NKCells
CD33+_Myeloid
CD14+_Monocytes
WholeBlood
Mean(24.3)
3xM
10xM

GSTT1

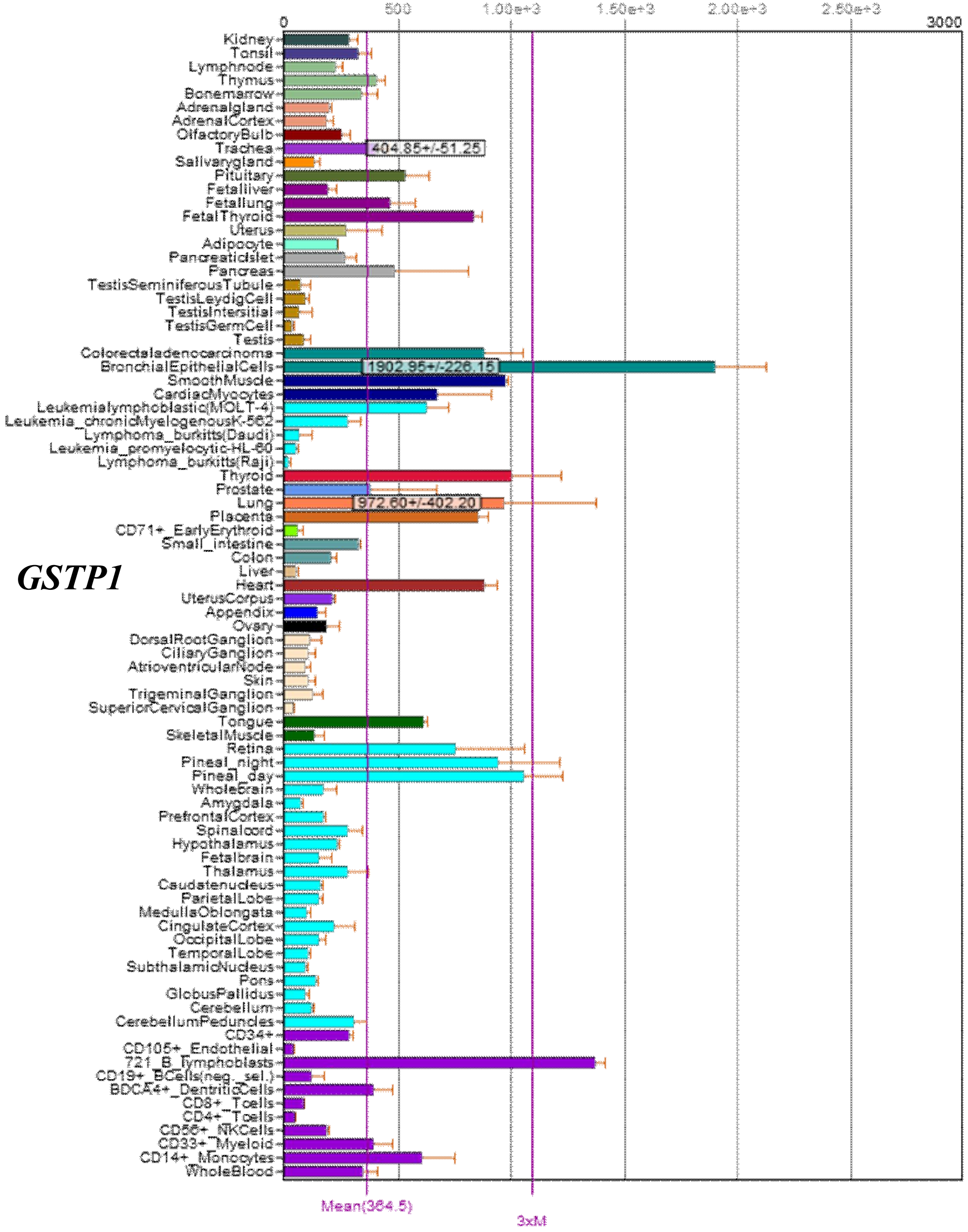

0
500
1.00e+3
1.50e+3
2.00e+3
2.50e+3
3000
Kidney
Tonsil
Lymphnode
Thymus
Bonemarrow
Adrenalgland
AdrenalCortex
OlfactoryBulb
Trachea
404.85+/-51.25
Salivarygland
Pituitary
Fetalliver
Fetallung
FetalThyroid
Uterus
Adipocyte
Pancreaticislet
Pancreas
TestisSeminiferousTubule
TestisLeydigCell
TestisIntersitial
TestisGermCell
Testis
Colorectaladenocarcinoma
BronchialEpithelialCells
1902.95+/-226.15
SmoothMuscle
CardiacMyocytes
Leukemialymphoblastic(MOLT-4)
Leukemia_chronicMyelogenousK-562
Lymphoma_burkitts(Daudi)
Leukemia_promyelocytic-HL-60
Lymphoma_burkitts(Raji)
Thyroid
Prostate
Lung
972.60+/-402.20
Placenta
CD71+_EarlyErythroid
Small_intestine
Colon
Liver
Heart
UterusCorpus
Appendix
Ovary
DorsalRootGanglion
CiliaryGanglion
AtrioventricularNode
Skin
TrigeminalGanglion
SuperiorCervicalGanglion
Tongue
SkeletalMuscle
Retina
Pineal_night
Pineal_day
Wholebrain
Amygdala
PrefrontalCortex
Spinalcord
Hypothalamus
Fetalbrain
Thalamus
Caudatenucleus
ParietalLobe
MedullaOblongata
CingulateCortex
OccipitalLobe
TemporalLobe
SubthalamicNucleus
Pons
GlobusPallidus
Cerebellum
CerebellumPeduncles
CD34+
CD105+_Endothelial
721_B_lymphoblasts
CD19+_BCells(neg._sel.)
BDCA4+_DentriticCells
CD8+_Tcells
CD4+_Tcells
CD56+_NKCells
CD33+_Myeloid
CD14+_Monocytes
WholeBlood
Mean(364.5)
3xM

GSTP1

CYP2E1

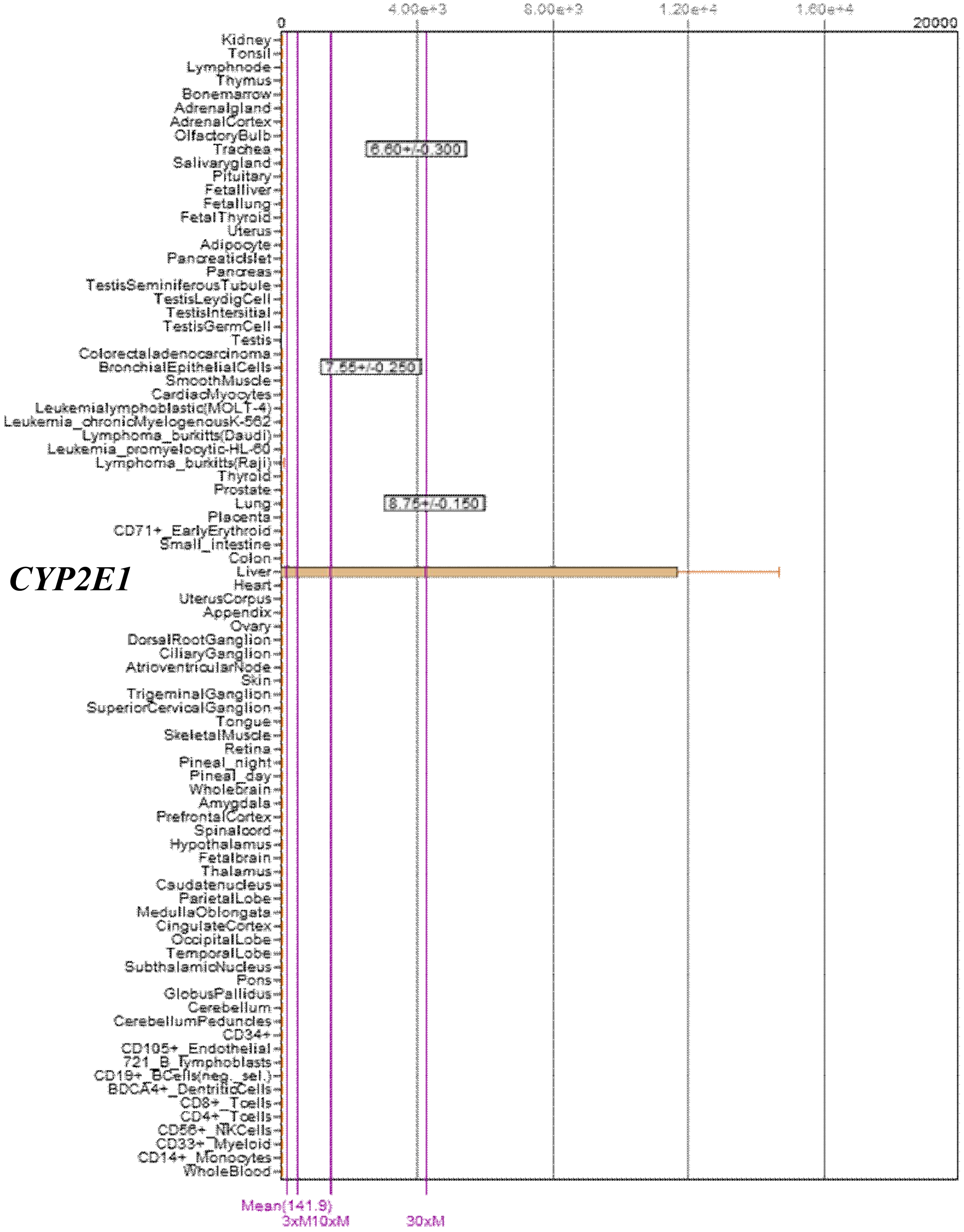

* indicates expression levels in cell types/tissues/organs of the respiratory system
